# Designing of a chimeric multiepitope vaccine against bancroftian lymphatic filariasis through immunoinformatics approaches

**DOI:** 10.1371/journal.pone.0310398

**Published:** 2024-09-19

**Authors:** Rehana Parvin, Md. Habib Ullah Masum, Jannatul Ferdous, Ahmad Abdullah Mahdeen, Md. Shafiqul Islam Khan

**Affiliations:** 1 Department of Pathology and Parasitology, Faculty of Veterinary Medicine, Chattogram Veterinary and Animal Sciences University (CVASU), Chattogram, Bangladesh; 2 Department of Genomics and Bioinformatics, Faculty of Biotechnology and Genetic Engineering, Chattogram Veterinary and Animal Sciences University (CVASU), Chattogram, Bangladesh; 3 Department of Obstetrics and Gynecology, Chittagong Medical College, Chittagong, Bangladesh; 4 Department of Microbiology, Noakhali Science and Technology University, Noakhali, Bangladesh; 5 Department of Cellular and Molecular Biology, Faculty of Biotechnology and Genetic Engineering, Chattogram Veterinary and Animal Sciences University (CVASU), Chattogram, Bangladesh; Chung-Ang University, REPUBLIC OF KOREA

## Abstract

The filarial worms of *Wuchereria bancrofti* are the primary cause of lymphatic filariasis (LF), a mosquito-borne disease among the neglected tropical parasitic diseases. Considering the global endemic consequences of the disease, there is a need to develop a successful vaccine candidate against LF. Using advanced immunoinformatics approaches, we designed two multiepitope vaccines targeting *W*. *bancrofti*’s glutathione S-transferase and thioredoxin. Therefore, we predicted several MHC-1, MHC-2, and B-cell epitopes from these proteins and mapped two vaccine candidates (V1 and V2). The vaccines were subsequently employed for physicochemical analysis, structural prediction and validation, docking and normal mode analysis, codon optimization, and immune simulation. The selected MHC-1, MHC-2, and B-cell epitopes were antigenic without allergenicity or toxicity. The designed vaccines were expected to be soluble, stable proteins under physiological conditions. Compared to V2, V1’s secondary and tertiary structures were simultaneously favorable, with Ramachandran plot analysis revealing 95.6% residues in favored areas. Subsequently, the molecular docking analysis indicated that the V1 had a high binding affinity for the TLR-2, TLR-4 and TLR-5, as suggested by the docking scores of -1248.7, -1038.5 and -1562.8, respectively. The NMA of these complexes further indicated their structural flexibility. Molecular dynamics simulations of V1-TLR complexes revealed V1-TLR-4 as the most stable, with the lowest free energy and minimal fluctuations, indicating the strongest binding affinity. The results of the codon optimization showed high levels of expression, with a favorable CAI score (<1.0). A three-dose vaccination analysis showed significant and persistent immunological responses, including adaptive and innate immune responses. The findings emphasize the potential of the V1 against *W*. *bancrofti*, but further validation is required through *in vitro*, *in vivo*, and clinical trials.

## 1. Introduction

Lymphatic filariasis (LF), also known as elephantiasis, is a mosquito-borne parasitic infection caused by the filarial nematodes *Wuchereria bancrofti*, *Brugia malayi*, and *Brugia timori* [1–3]. According to the World Health Organization (WHO), 90% of LF cases are associated with *W*. *bancrofti* infection, which is also known as bancroftian lymphatic filariasis (BLF) [2, 4, 5]. LF, a highly significant form of filarial infection, primarily affects the human lymphatic system and can persist in the body for 5–10 years by evading the host’s defense mechanisms [6]. Based on a survey released in 2008, LF is endemic in over 83 countries worldwide [7–9]. Southeast Asia—including Bangladesh, India, Nigeria, and Indonesia—has become the global hub for LF, accounting for 70% of cases [10, 11]. As reported earlier, LF has already infected 120 million people and poses a risk to over 1.2 billion [12]. Global Programme to Eliminate LF (GPELF) was initiated in 2000 to eliminate LF by 2020, and it was authorized by the World Health Assembly Resolution 50.29 (WHA 50.29) [12]. A recent WHO report indicates that 882.5 million people across 44 countries remain at risk for LF [2, 13].

The causative agent of LF are primarily transmitted by vector mosquitoes from the *Anopheles*, *Aedes*, *Culex*, and *Mansonia* genera [14, 15], with variations depending on the region [8]. In India, *Culex* spp. is the primary carrier of the parasite, which is responsible for 99.4% of LF cases [16–18]. However, in other areas, including the island of New Guinea, Southern Asia, and West Africa, *Anopheles* species play a critical role in the spread of the disease [7–9]. Several health issues have been seen as a consequence of this disease, including hydrocele, lymphedema, elephantiasis, chyluria, chylous diarrhea, chylorrhagia [19, 20] and fatal chronic conditions [9, 21]. In principle, LF is the second leading cause of long-term disability, after mental disorders [22, 23], with approximately 40 million people either disabled or incapacitated by the disease [12, 24, 25].

Mass drug administration (MDA) has long been employed to fight LF, utilizing diethylcarbamazine citrate (DEC) as well as a combination of DEC and albendazole [12, 26]. However, the disease has yet to be eradicated due to several imperfections with the current medications, including poor absorption, toxic side effects, limited effectiveness against adult parasites, and low absorption [1]. Therefore, alternative strategies like pre-immunization or vaccination are required to eradicate the occurrences of LF [27]. And these approaches need to be effective against the parasite throughout all or most stages of its life cycle [22, 23]. The conventional approach for developing vaccine candidates and triggering the appropriate antibody-mediated response is expensive, time-consuming, and labor-intensive. On the other hand, vaccines produced by immunoinformatics approaches address the drawbacks of traditional approaches. These approaches use pathogen genomic data, such as the epitope map, to develop vaccines instead of using in vitro culture of parasites [28, 29].

The presence of the redox regulatory system in the filarial parasite is one of the most important factors behind its survival within the host body. Hence, antioxidants such as glutathione S-transferase and thioredoxin are the key redox-active proteins essential for their survival in the host body [30, 31]. Where these proteins facilitate the parasite to survive and withstand oxidative stress of the host defense mechanism, including reactive oxygen species (ROS) components [24, 32]. Both proteins play an essential role in maintaining redox homeostasis by reducing hydrogen peroxide (H2O2) [24, 31], catalyzing the dismutation of the superoxide radical as a detoxification process, and protecting the parasite’s membranes from damage by phospholipid peroxidation [24, 30]. However, both proteins are known to be expressed throughout the filarial parasite’s lifespan, so generating a vaccine against it could effectively combat all stages of filarial parasite development [24, 31].

This study aims to design a chimeric multiepitope vaccine against *W*. *bancrofti* that could effectively combat LF. We employed advanced immuninformatics approaches to predict epitopes specific to T cells (major histocompatibility complex-1), cytotoxic T lymphocytes (major histocompatibility complex-2), and B-cells based on the amino acid sequences of two proteins: glutathione S-transferase and thioredoxin. The epitopes were linked using four linkers (EAAAK, AYY, AK, and KFER). Finally, two immune stimulatory adjuvants, heparin-binding hemagglutinin (HBHA) and 50S ribosomal protein L7/L12 (bl12) were used in the vaccine map and linked to the remaining epitope to construct the final vaccine map.

## 2. Materials and methods

### 2.1 Sequence retrieval

**Fig 1:**
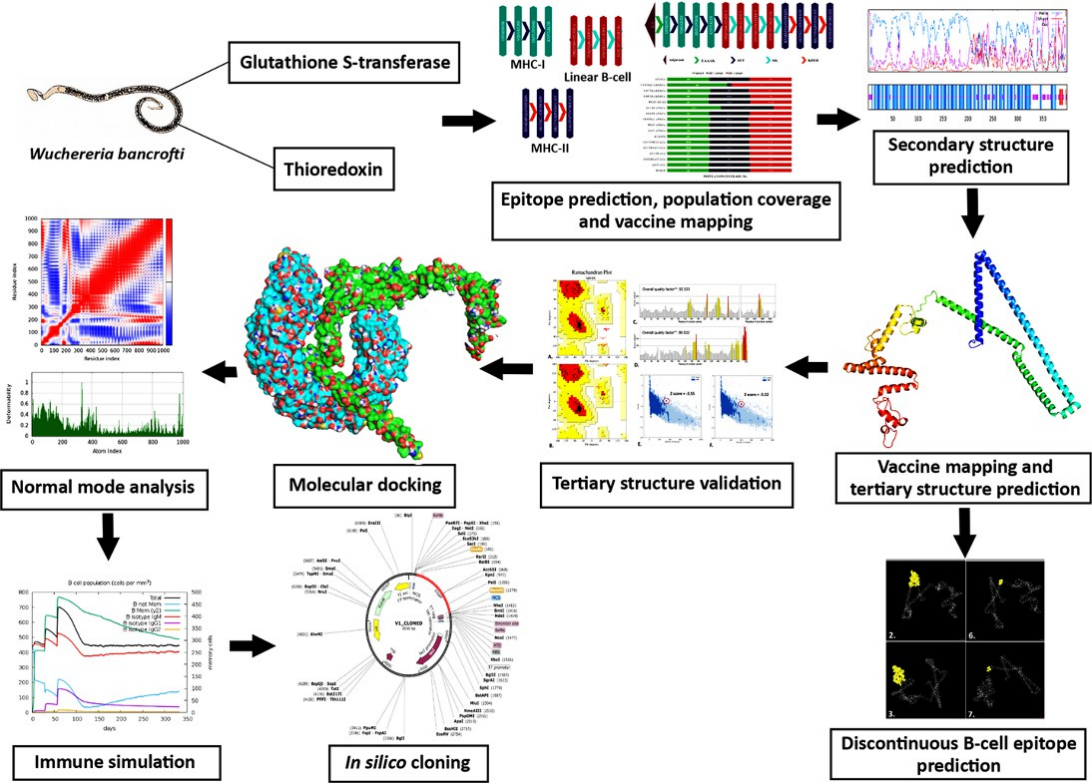
The overview of the mRNA vaccine design.

The National Centre for Biotechnology Information (NCBI) protein database was accessed to acquire the amino acid sequences of *W*. *bancrofti*’s two primary proteins, glutathione S-transferase and thioredoxin [33]. The protein codes were saved in the FASTA file, which was then used to construct the vaccine. **Fig 1** shows an outline of the work.

### **2.2 Prediction of MHC-1, MHC-2 and linear B-cell** epitope

The MHC-1 binding epitopes of the retrieved proteins, including glutathione S-transferase and thioredoxin, were predicted by the MHC-1 Binding Predictions tool of the Immune Epitope Database (IEDB) [34, 35] and Net-MHC 4.0 (http://www.cbs.dtu.dk/services/NetMHC/) server [36, 37]. Likewise, the MHC-2 binding epitopes were also predicted using the IEDB’s MHC-2 Binding Predictions tool [34, 35] (http://tools.iedb.org/mhcii/) and NetMHCIIpan 4.0 server (http://www.cbs.dtu.dk/services/NetMHCIIpan/) [35, 38]. Further, the linear B-cell epitopes of the protein of interest were predicted by the IEDB’s Antibody Epitope Prediction tool and the ABCpred (https://webs.iiitd.edu.in/raghava/abcpred/ABC_submission.html) server [39]. The selected epitopes were further evaluated for antigenicity, allergenicity and toxicity by VaxiJen 2.0 [40], AllerTOP v. 2.0 [41] and ToxinPred server [42], respectively.

### 2.3 Population coverage

Variation in ethnicity and geography affects the frequency of individual HLA alleles [43, 44]. Therefore, the IEDB population coverage tool was employed to assess the potential population coverage of the designed vaccines (http://tools.iedb.org/population/) [45]. For this analysis, we used the chosen MHC-1, MHC-2 epitopes and their corresponding alleles (both combinedly and individually). We also highlighted the specific genetic variants that are found globally.

### 2.4 Vaccine mapping

The mapping of vaccine 1 (V1) and vaccine 2 (V2) was executed by utilizing highly prioritized epitopes from the selected proteins (glutathione S-transferase and thioredoxin). Heparin-binding hemagglutinin (HBHA) was used as an immunostimulatory adjuvant in V1, while the 50S ribosomal protein L7/L12 (bl12) was used in V2. Appropriate linkers, including EAAAK, AYY, AK, and KFER, were employed in the final vaccine mapping process.

### 2.5 Post-vaccine mapping analysis

After mapping the vaccines, the physicochemical properties, solubility, allergenicity, and antigenicity were analyzed. The properties examined included molecular weight, total amino acid count, instability index, aliphatic index, isoelectric point (pI), grand average of hydropathicity (GRAVY), the number of positively and negatively charged residues, and the total number of atoms. These characteristics were predicted using the Expasy ProtParam server (http://web.expasy.org/protparam/) [46]. Subsequently, the SOLpro server was used to calculate the solubility of the vaccines (https://scratch.proteomics.ics.uci.edu/) [47]. Any allergenic properties of the vaccines were also evaluated by AllerTOP v. 2.0 server (https://www.ddg-pharmfac.net/AllerTOP/) [41] and AlgPred (https://webs.iiitd.edu.in/raghava/algpred/submission.html) [48]. Finally, the antigenic characteristics of the vaccines were also assessed by SCRATCH (http://scratch.proteomics.ics.uci.edu) [49] and VaxiJen 2.0 server [40].

### 2.6 Secondary and tertiary structure prediction, refinement, and validation

The secondary structures of the vaccines were predicted by the GOR4, and SOPMA server [16, 17, 50, 51], whereas the 3Dpro program of the SCRATCH suite was utilized for the tertiary (3D) structure prediction [52]. The 3D structures of the vaccines were further applied for structural refinement by the GalaxyWEB server [53]. Afterward, the structural validations of the vaccines were carried out by the SAVES v6.0 server (https://saves.mbi.ucla.edu/), which defines the stereochemical quality of the predicted vaccine models through Ramachandran plot and ERRAT analysis [54–57]. We utilized the ProSA-web server to assess the precision of the predicted vaccines’ 3D model structure. The server offers a Z-score to evaluate the precision and potential flaws of the predicted 3D model structures [58, 59]. Here, a lower Z-score indicates a higher-quality protein model [59].

### 2.7 Prediction of discontinuous B-cell epitopes

Discontinuous B-cell epitopes are essential for effective vaccine design because they naturally mimic the structure of pathogens, leading to a more robust immune response. These epitopes comprise non-consecutive amino acids arranged into a three-dimensional shape, crucial for precise antibody attachment [60]. Hence, the discontinuous B-cell epitopes of the vaccines were predicted by the Ellipro (http://tools.iedb.org/ellipro/) server [61]. The server (http://tool s.iedb.org/ellipro/) predicts potent discontinuous B-cell epitopes via a combination of three algorithms [61].

### 2.8 Molecular docking study

Considering the molecular docking study, the CLUSPRO 2.0 (cluspro.bu.edu/login.php) server was utilized to identify the binding affinities of the vaccines towards the human TLRs (Toll-like receptors) [62–65]. The tertiary structures of the TLR-2 (Toll-like receptor 2) (PDB: 2Z7X), TLR-4 (Toll-like receptor 4) (PDB: 3FXI) and TLR-5 (Toll-like receptor 5) (PDB: 3J0A) were obtained from the Protein Data Bank (PDB) database (www.rcsb.org) before the docking study [66–68]. The docked complexes were then visualized by the PyMOL, while the intermolecular interactions were analyzed by the PDBsum server [69].

### 2.9 Normal mode analysis

The functional movements of macromolecules can be characterized by the NMA [70–72]. Consequently, the functional and macromolecular movements of the "V1-TLR-2", "V1-TLR-4", "V1-TLR-5", "V2-TLR-2", "V2-TLR-4", and "V2-TLR-5" complexes’ were executed by the iMODS server [72]. The server offers a variety of substantial motion configurations, including affine-model arrows, vector fields, and modal animations. Hence, it calculates various properties, such as mobility (B-factor), deformability, eigenvalues, covariance map, and linkage matrix. [72]. The B-factor quantifies how atoms deviate from their equilibrium position in a structure. The deformability plot is a visual representation of the flexibility of proteins, specifically focusing on coil or domain linkers. Moreover, the eigenvalue indicates the complex’s stability, with larger values indicating a greater degree of stability [71, 72].

### 2.10 Molecular dynamic simulation

After evaluating the quality of the tertiary structure and analyzing the findings of molecular docking and normal mode analysis, we selected the V1 and its complexes for further investigations. A molecular dynamic simulation was conducted to assess the stability of the "V1-TLR-2", "V1-TLR-4", and "V1-TLR-5" complexes in a simulated physiological environment utilizing a virtual model building with energy refinement (AMBER 18) [73], and ff19SB force field [74] with OPC water model [75, 76]. Three-layer solvation was achieved by utilizing the octahedron box shape with the V1, "V1-TLR-2", "V1-TLR-4", and "V1-TLR-5" complexes, ensuring their positions were at least 12Å away from the edge of the water-filled box [77]. The system was neutralized with the concourse of Amber’s "tleap" package by adding Na+ and Cl− counter ions. The systems were reduced at 500 steepest descent cycles and 1000 conjugate gradient steps to remove constraint atoms. To achieve conformational stability, the systems were heated for fifty picoseconds (ps) utilizing langevin dynamics and kept the temperature at 300 K throughout the experiment. Subsequently, the system was stabilized for 5 nanoseconds (ns) under controlled temperature and pressure conditions using isotropic position scaling. The simulation was conducted throughout 100 nanoseconds using the SHAKE and particle-mesh Ewald (PME) strategies in the pmemd.cuda [78, 79]. A cut-off radius of 10Å was used to determine non-bond contacts in long-term interactions.

### 2.11 Molecular mechanics with generalised born and surface area solvation (MMGBSA)

Using the molecular mechanics and the generalized born approach, the free binding energies (ΔTOTAL) associated with the "V1-TLR-2", "V1-TLR-4", and "V1-TLR-5" were evaluated. During this analysis, different intermolecular interactions were analyzed, including van der Waals forces (ΔVDWAALS), electrostatic interactions (ΔEEL), as well as polar (ΔEGB) and non-polar (ΔESURF) components [80–82]. However, the HawkDock server was utilized for the MMGBSA analysis [80–82], where the accuracy of the server was reported to be between 80–95% for the crystal and predicted structures [83].

### 2.12 Molecular mechanics poisson-boltzmann surface area (MMPBSA)

The MMPBSA.py package of AMBER 18 program was utilized to execute the MMPBSA for the "V1-TLR-2", "V1-TLR-4", and "V1-TLR-5" complexes [84]. This molecular mechanic approach, like MMGBSA, evaluates several intermolecular interactions, including binding contacts (ΔTOTAL), electrostatic interactions (ΔEEL), van der Waals forces (ΔVDWAALS), polar (ΔEGB), and non-polar (ΔESURF) components [80–82].

### 2.13 Codon optimization and *in silico* cloning

The Java Codon Adaptation Tool (JCat) server (http://www.jcat.de/Start.jsp) was used to optimize the codons of the vaccine candidate, employing the *E*. *coli* strain K12. To assess a protein’s expression level, the server calculates its GC contents and codon adaptation index (CAI). On the other hand, a score of ≥ 0.8 is accepted as good, and ≥ 1.0 is the best CAI value. However, the permissible GC contents can vary from 30% to 70% [85, 86]. Afterward, the optimized gene sequence of the V1 was ligated to the *E*. *Coli* plasmid vector pET-28a(+), with restriction sites EcoRI and BamHI at N and C-terminals of the corresponding vaccine sequence. Finally, using the SnapGene program (https://www.snapgene.com/free-trial/), the optimized V1 sequence was transferred into the plasmid vector pET-28a(+).

### 2.14 Immune simulation

The immune simulations of the V1 were assessed using the C-ImmSim server (https://kraken.iac.rm.cnr.it/C-IMMSIM/) [87]. The simulation was assessed for different HLA populations, including Europe (HLA-A*01:01, HLA-A*02:01, HLA-B*07:02, HLA-B*51:01, HLA-DRB1*15:01, and HLA-DRB1*07:01), South, West and Central Asia (HLA-A*02:01, HLA-A*11:01, HLA-B*51:01, HLA-B*07:02 and HLA-DRB1*07:01), North and Central America (HLA-A*02:01, HLA-A*24:02, HLA-B*15:01, HLA-B*51:01, HLA-DRB1*15:01, and HLA-DRB1*07:01), Oceania (HLA-A*24:02, HLA-A*11:01, HLA-B*40:01, HLA-B*15:01, HLA-B*51:01, HLA-DRB1*15:01, and HLA-DRB1*07:01), North and East Asia (HLA-A*02:01, HLA-A*24:02, HLA-B*15:01, HLA-B*51:01, and HLA-DRB1*15:01), South America (HLA-A*02:01, HLA-A*24:02, HLA-B*15:01, and HLA-DRB1*15:01). To ensure proper immunization, the V1 was designed with three doses of a regime that will be administered at four-week intervals. The simulation was run using the server’s default parameters, with time steps set to 1, 84, and 168. Finally, the simulation steps and volume were set to 50 and 1000, respectively. The server’s default configuration without lipopolysaccharides (LPS) was also set to the random seed.

## 3. Results

### 3.1 Sequence retrieval

The amino acid sequences of *W*. *bancrofti*’s two primary proteins, glutathione S-transferase (accession number: AAO45827.1) and thioredoxin (accession number: EJW80481.1), were obtained from the National Center for Biotechnology Information (NCBI) protein database. These protein sequences were saved in FASTA format and subsequently used for vaccine design.

### 3.2 Prediction of MHC-1, MHC-2 and linear B-cell epitope

A total of 12 epitopes, including four MHC-1, four MHC-2 and four B-cell epitopes, were selected from the retrieved proteins. The threshold value was set at <2.0 while selecting the MHC-1 and MHC-2 epitopes. However, 26 HLA alleles (HLA-B*44:02, HLA-B*15:01, HLA-A*68:01, HLA-B*57:01, HLA-A*03:01, HLA-A*02:06, HLA-A*30:02, HLA-A*24:02, HLA-A*23:01, HLA-B*07:02, HLA-A*31:01, HLA-B*40:01, HLA-B*08:01, HLA-A*11:01, HLA-A*30:01, HLA-A*33:01, HLA-A*32:01, HLA-A*26:01, HLA-B*53:01, HLA-B*44:03, HLA-B*35:01, HLA-A*01:01, HLA-A*68:02, HLA-B*58:01, HLA-B*51:01, HLA-A*02:01 and HLA-A*02:03) were chosen for MHC-1 and 7 HLA-DRB alleles (HLA-DRB4*01:01, HLA-DRB3*01:01, HLA-DRB5*01:01, HLA-DRB1*15:01, HLA-DRB1*03:01, HLA-DRB3*02:02 and HLA-DRB1*07:01) were chosen for MHC-2 epitope prediction. However, for the final vaccine construct, the most probable antigenic, non-allergenic, and non-toxic MHC-1, MHC-2, and B-cell epitopes were selected from each protein **(Table 1)**.

**Table 1:** List of selected MHC-1, MHC-2, and B-cell epitopes with their antigenicity, allergenicity, and toxicity assessment.

Protein	Epitope	Peptide	Perce-ntile rank	Allergenicity	Antigenicity	Toxicity
Glutathione S-transferase	MHC-1	DTEKDSYIK	1.1	Probable non-allergen	1.8558 (Probable ANTIGEN)	Non-toxin
		VQSGAILRH	0.55	Probable non-allergen	0.6948 (Probable ANTIGEN)	Non-toxin
	MHC-2	DHQIVQSGAILRHLA	0.47	Probable non-allergen	0.5530 (Probable ANTIGEN)	Non-toxin
		KDSYIKDILPVELAK	1.3	Probable non-allergen	1.0979 (Probable ANTIGEN)	Non-toxin
	B-cell	KYAKMIYQAYDTEKDS	0.82	Probable non-allergen	0.9893 (Probable ANTIGEN)	Non-toxin
		AKFEKLLATRDDGKNF	0.72	Probable non-allergen	0.7721 (Probable ANTIGEN).	Non-toxin
Thioredoxin	MHC-1	HMYLFPDMK	0.16	Probable non-allergen	0.8051 (Probable ANTIGEN)	Non-toxin
		KYPTLKLFR	0.15	Probable non-allergen	1.0880 (Probable ANTIGEN)	Non-toxin
	MHC-2	DNYRKVASLLRDDCV	0.12	Probable non-allergen	0.6148 (Probable ANTIGEN)	Non-toxin
		ADALAVFIDKQLVSG	0.23	Probable non-allergen	0.9933 (Probable ANTIGEN)	Non-toxin
	B-cell	KKEYRGQRSADALAVF	0.89	Probable non-allergen	0.8089 (Probable ANTIGEN)	Non-toxin
		NAVAWATVDCDREADI	0.83	Probable non-allergen	0.8901 (Probable ANTIGEN)	Non-toxin

### 3.3 Population coverage

**Fig 2:**
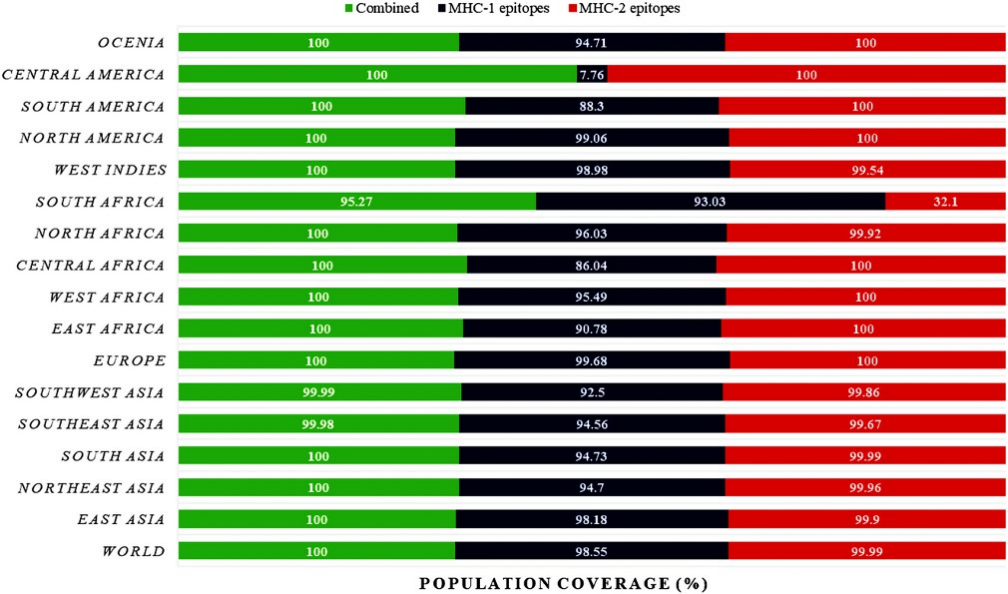
The population coverage of the selected MHC-1 and MHC-2 epitopes.

The total population coverage is consistently at or nearly 100% worldwide **(Fig 2)**. Most regions demonstrate substantial coverage for MHC-1 and MHC-2 epitopes, suggesting strong overall population coverage. However, the overall MHC-1 epitope coverage is high, except for 7.76% in Central America, and MHC-2 epitope coverage exists high, with a distinction of 32.1% in South Africa. However, the population coverage showed a worldwide distribution of specific genetic variations on various continents, both individually and in combination **(Fig 2)**.

### 3.4 Vaccine mapping

**Fig 3:**
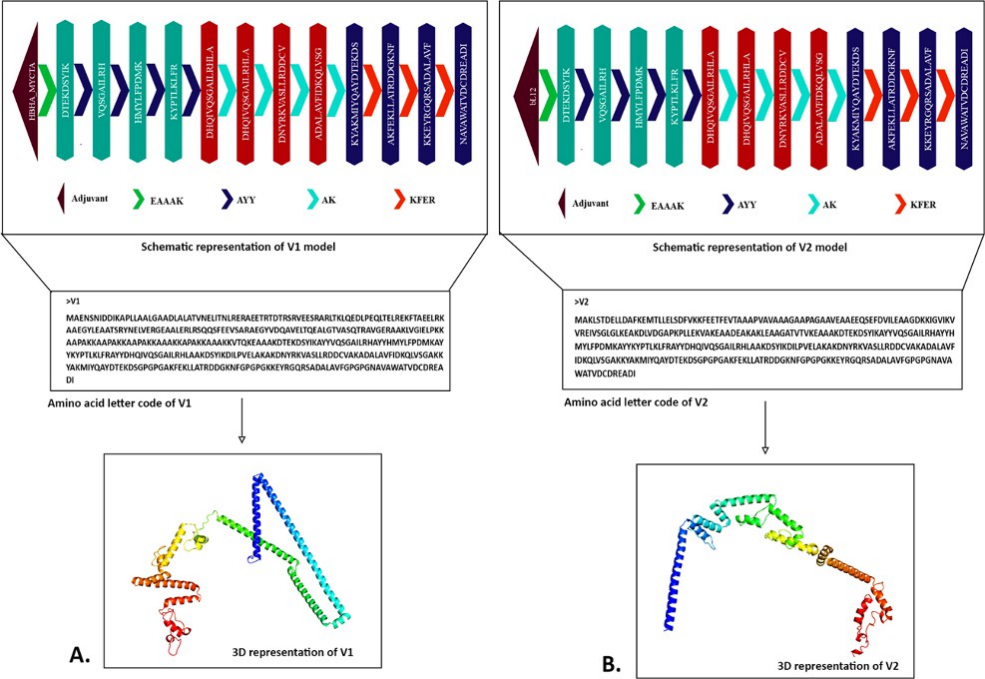
The mapping of designed V1 **(A)** and V2 **(B)** against *W*. *bancrofti*. The schematic presentation of the vaccines containing adjuvant (dark maroon), epitopes (MHC-1-dark cyan MHC-2-maroon, linear B-cell-dark purple), and linkers (EAAAK- light green, AYY-purple, AK-cyan, KFER-red).

Adjuvants play a vital role in vaccine development by enhancing the vaccine’s ability to trigger an immune response, increasing its stability, and prolonging its effectiveness [88]. To activate the innate and adaptive immune systems, they require a carrier that contains potent immunostimulatory adjuvants [89]. The epitopes were conjugated using two adjuvants, HBHA and bl12, and were used in the V1 and V2, respectively. Linkers are necessary for the vaccine’s design to replicate the immunogen’s ability to function as a separate immunogen and produce higher antibody concentrations than a single immunogen [90]. Four linkers, EAAAK, AYY, AK, and KFER, were used to join the selected adjuvants and epitopes in the final vaccine structures **(Fig 3)**.

### 3.5 Post-vaccine mapping analysis

The physicochemical properties of the vaccines (V1 and V2) were obtained using the ExPASy ProtParam server. According to the server, V1 consists of 299 amino acids, while V2 is made up of 330 amino acids. The molecular weights of the V1 and V2 were predicted to be 21,534.46 Da and 1,344.48 Da, respectively. The isoelectric points (pI) were calculated to be 9.17 for V1 and 4.59 for V2, indicating that V1 is basic (pH > 7) while V2 is acidic (pH < 7). Furthermore, the predicted GRAVY scores were -0.593 for V1 and 0.180 for V2, suggesting that both vaccines are water-soluble (**Table 2**). The server also confirmed that these vaccines are well stable proteins, with instability indices of 46.67 for V1 and 19.91 for V2. Meanwhile, the aliphatic index was predicted to be 84.67 for V1 and 103.77 for V2 (**Table 2**). However, the V1 (32 and 36) has more negatively and positively charged residues than the V2 (25 and 16). Additionally, both vaccines were effectively soluble when expressed in *E*. *coli*, as confirmed by the SOLpro server. Moreover, both the V1 and V2 were predicted to be non-allergenic. Finally, the VaxiJen 2.0 and SCRATCH servers indicated that the vaccines are likely to function as antigens with high antigenic scores (**Table 2**).

**Table 2:** The physicochemical and immunological properties of the V1 and V2.

Physicochemical properties	V1	V2
Molecular Weight (Da)	21534.46	1344.48
Number of amino acids	199	130
Theoretical pI	9.17	4.59
Grand average of hydropathy (GRAVY)	-0.593	0.180
Instability index	46.67	19.91
Aliphatic index	84.67	103.77
Total number of negatively charged residues (Asp+Glu)	32	25
Total number of positively charged residues (Arg+Lys)	36	16
Number of atoms	3083	1935
Solubility (SOLpro)	Soluble protein	Soluble protein
Allergenicity (AlgPred /AllerTop)	Probable non-allergen	Probable non-allergen
Antigenicity (VaxiJen 2.0/ SCRATCH)	Probable Antigen	Probable Antigen

### 3.6 Secondary and tertiary structure prediction, refinement and validation

The secondary structures of the V1 and V2 were predicted using the GOR4 and SOPMA servers. For V1, the GOR4 server indicated 78.70% alpha helix, 3.01% extended strands (beta sheet), and 18.30% random coil structure. Meanwhile, the SOPMA server predicted 70.18% alpha helix, 7.27% extended strands, and 19.55% random coil for V1. Notably, SOPMA predicted 3.01% beta-turn structures in V1, which were not identified by the GOR4 (**Table 3 and S1 Fig**). For V2, the GOR4 server indicated 67.88% alpha helix, 6.97% extended strands (beta sheet), and 25.15% random coil structure. In contrast, the SOPMA server predicted 59.70% alpha helix, 10.91% extended strands, and 21.52% random coil for V2. Additionally, the SOPMA predicted 7.88% beta-turn structures in V2, which were not detected by the GOR4 (**Table 3 and S1 Fig**).

**Table 3:** Secondary structure predictions and comparative assessment of V1 and V2 using GOR4 and SOPMA.

Properties	V1		V2	
	GOR4	SOPMA	GOR4	SOPMA
Alpha helix	78.70% (314)	70.18% (280)	67.88% (224)	59.70% (197)
Extended strand	3.01% (12)	7.27% (29)	6.97% (23)	10.91% (36)
Beta turn	0.00% (0)	3.01% (12)	0.00% (00)	7.88% (26)
Random coil	18.30% (73)	19.55% (78)	25.15% (83)	21.52% (71)

**Fig 4:**
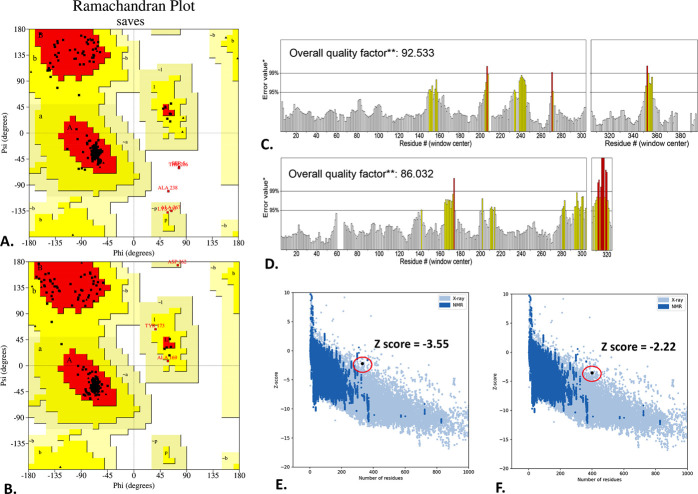
Structural validation of the V1 and V2 predicted by I-TASSER. The refined 3D structures are evaluated through the Ramachandran plot (**A, B**), ERRAT (**C, D**), and Z score (**E, F**).

The SCRATCH’s 3Dpro program predicted two reliable 3D structures for the V1 and V2. The refined 3D model of V1 was later obtained from the GalaxyWEB server, with an RMSD of 0.301, a MolProbity score of 2.783, and 94.8% of residues in Ramachandran’s favored region. Meanwhile, the refined 3D model of the V2 represented RMSD, MolProbity score, and Ramachandran’s favored region of 0.439, 2.731, and 93.4%, respectively, accomplishing both proteins with well-stable 3D structures. However, the SAVES’s Ramachandran plot depicted that 95.6% of amino acid residues of the refined V1 are in the most favored region, while 3.8% are in the additional allowed and generously allowed region (**Fig 4A**). On the other hand, the refined V2 has 96.9% amino acid residues in the most favored region, whereas 9.6% are in the additional and generously allowed region (**Fig 4B**). The ERRAT score for the refined V1 model was 92.533, whereas the V2 model received an ERRAT score of 86.032 (**Fig 4C and 4D**). Additionally, the ProSA server confirmed the energy-minimized models’ quality by assigning Z-scores of -3.55 for V1 and -2.22 for V2 **(Fig 4E and 4F).** A lower z-score indicates a higher-quality protein model; hence, the model quality of the V1 is better than the V2 model quality [59].

### 3.7 Prediction of discontinuous B-cell epitopes

**Fig 5:**
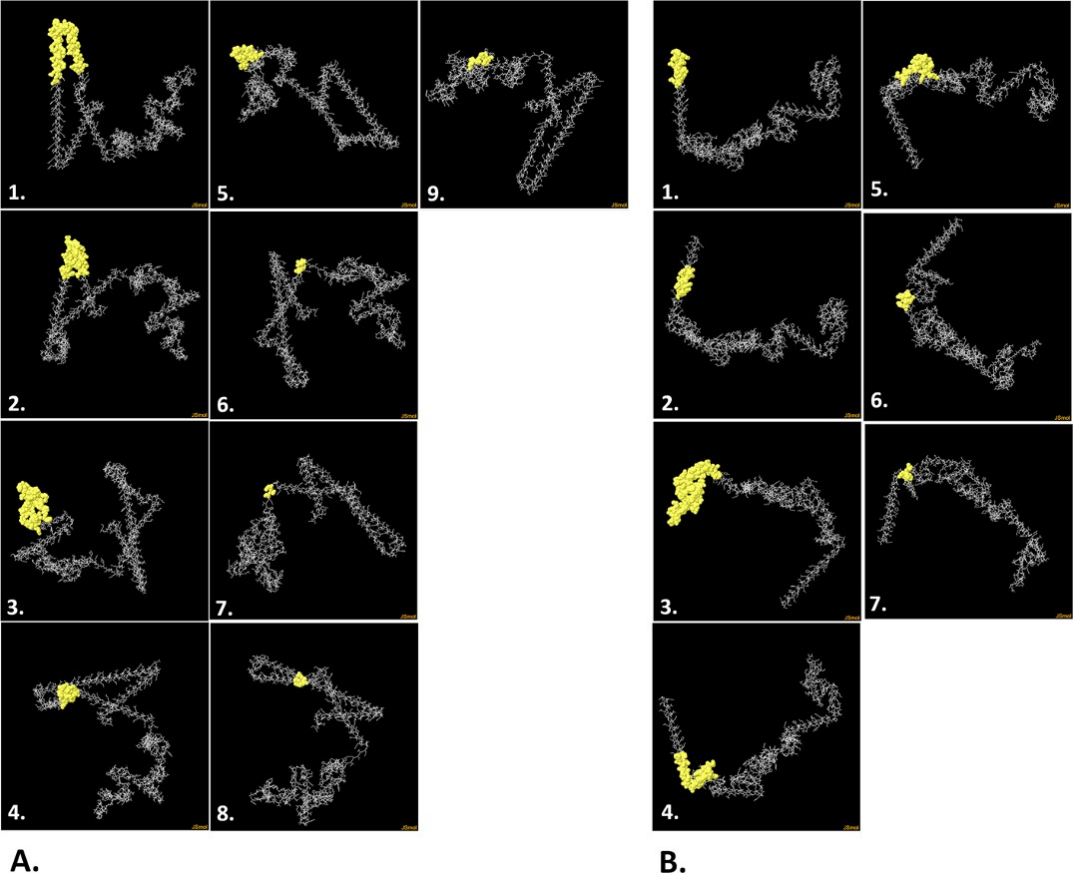
The discontinuous B-cell epitopes of the V1 (**A**) and V2 (**B**) predicted by Ellipro server. The discontinuous B-cell epitopes are presented as yellow-color surfaces while the whole vaccines are depicted with grey sticks.

The Ellipro server predicted 196 discontinuous B-cell epitopes for V1 and 168 for V2 (**Fig 5 and S1 Table**). V1 epitopes score between 0.509 and 0.825, depending on the number of residues, while V2 epitopes score between 0.513 and 0.975 (**S1 Table**). The higher the score, the more likely the location in the query will function as a conformational B-cell epitope. As a result, these vaccination sites might behave as B-cell epitopes, potentially producing antibodies (**Fig 5**).

### 3.8 Molecular docking study

The docking analyses between the vaccines (V1 and V2) and human TLR-2, TLR-4 and TLR-5 were performed using the CLUSPRO 2.0 server. According to the server, the lowest energy scores of the "V1-TLR-2", "V1-TLR-4", and "V1-TLR-5" complexes were predicted to be -1248.7, -1038.5 and -1562.8, respectively. On the other hand, the lowest energy scores of the "V2-TLR-2", "V2-TLR-4" and "V2-TLR-5" complexes were predicted to be -1004.2, -1078.5 and -1354.5 respectively. Further, PyMOL and PDBsum tools visualized and analyzed these docked complexes.

**Fig 6:**
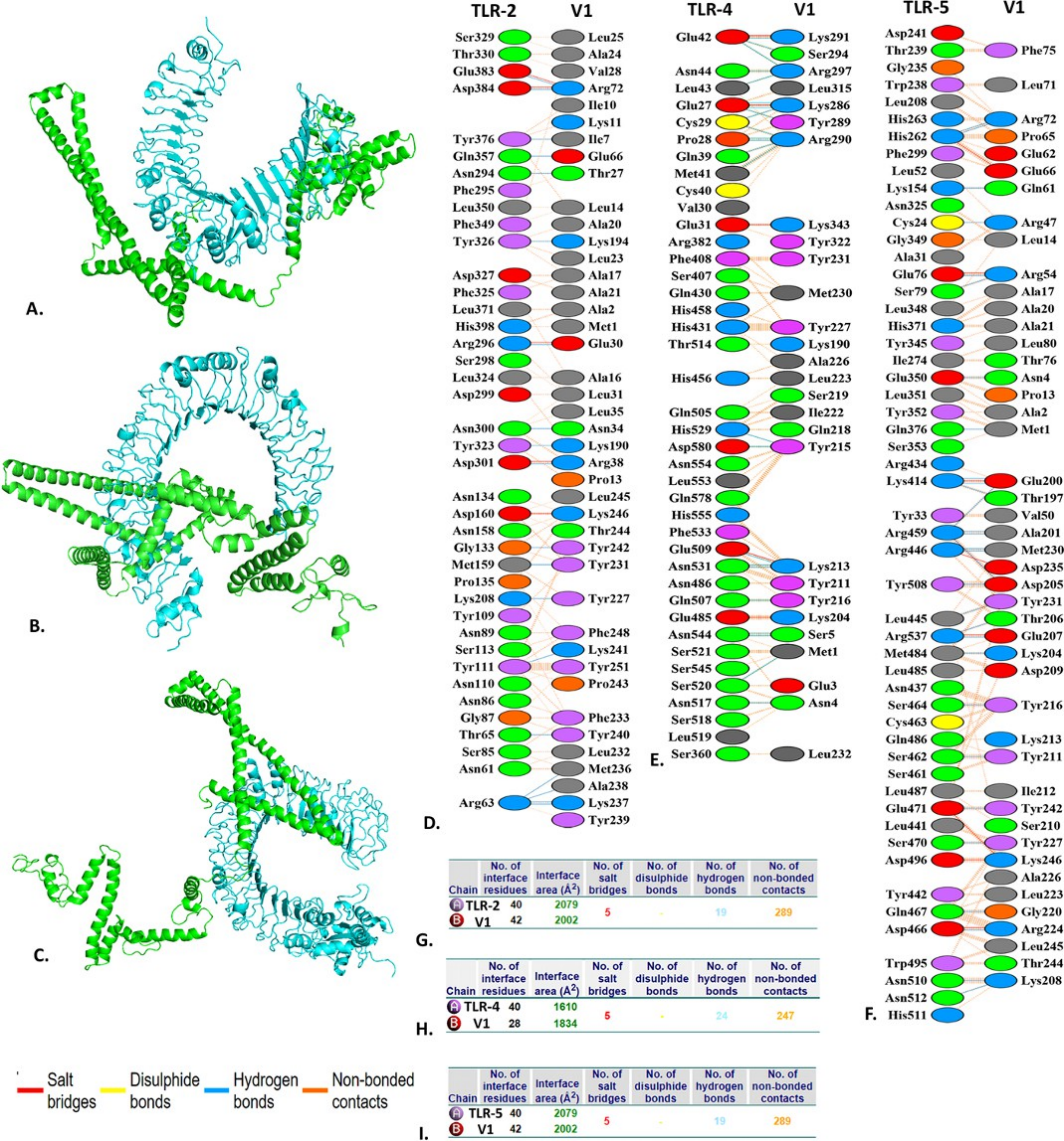
The docking analysis of the "V1-TLR-2" (A), "V1-TLR-4" (B), and "V1-TLR-5" (C) complexes. The intermolecular interactions between the "V1-TLR-2" (D, G), "V1-TLR-4" (E, H), and "V1-TLR-5" (F, I) complexes are represented as different color codes. The cyan and green colors represent the TLRs and V1, respectively. The red, yellow, blue, and orange colors represent the salt bridges, disulfide, hydrogen, and non-bonded contacts, respectively.

Based on the PDBsum, the "V1-TLR-2" complex has 5 hydrogen bonds, 19 salt bridges, and 289 non-bond interactions, whereas the "V1-TLR-4" complex has 4 hydrogen bonds, 2 salt bridges, and 257 non-bond interactions. Moreover, the "V1-TLR-5" has a similar number of hydrogen bonds, salt bridges and non-bond interactions to the "V1-TLR-2" **(Table 4 and Fig 6)**. Regarding the "V2-TLR-2", the server predicted 15 hydrogen bonds, 6 salt bridges and 155 non-bond interactions, whereas, it predicted 15 hydrogen bonds, 6 salt bridges and 155 non-bond interactions in the "V2-TLR-4". However, maximum intermolecular interactions were found in the "V1-TLR-5", including 24 hydrogen bonds, 10 salt bridges and 328 non-bond interactions. Interestingly, none of these complexes contain disulfide bonds **(Table 4 and S2 Fig)**.

**Table 4:** The docking scores and interactions between the vaccine-receptor complexes.

Vaccine	Complex	Lowest energy	Interactions between the vaccine and TLRs			
			Salt bridges	Disulphide bonds	Hydrogen bonds	Non-bonded contacts
V1	V1-TLR-2	-1248.7	5	-	19	289
	V1-TLR-4	-1038.5	5	-	2	257
	V1-TLR-5	-1562.8	5	-	19	289
V2	V2-TLR-2	-1004.2	6	-	15	155
	V2-TLR-4	-1078.5	5	-	14	169
	V2-TLR-5	-1354.5	10	-	24	328

### 3.9 Normal mode analysis

**Fig 7:**
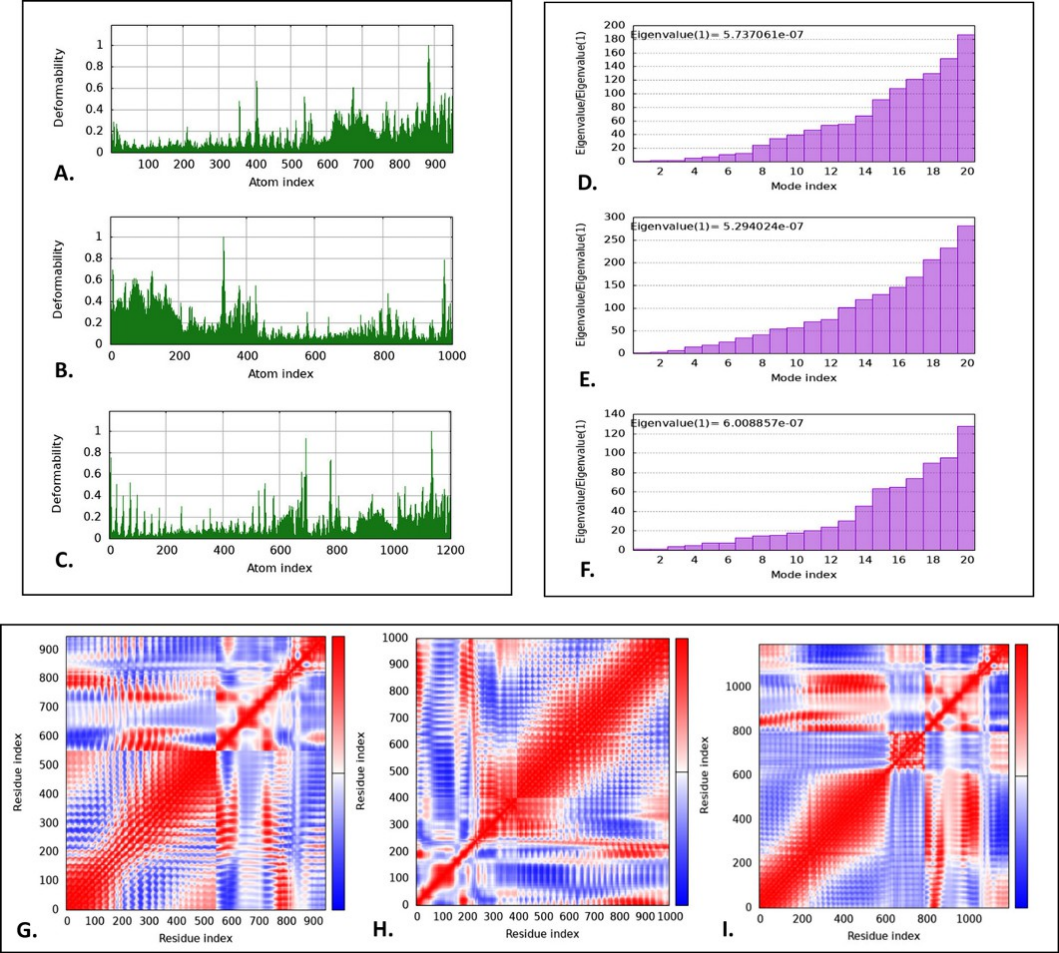
The normal mode analysis of the "V1-TLR-2", "V1-TLR-4", "V1-TLR-5", "V2-TLR-2", "V2-TLR-4", and "V2-TLR-5" docked complex. The illustration depicted the deformability plots (**A, B, C**), eigenvalue (**D, E, F**), and co-variance map (**G, H, I**).

The iMODS server was employed to perform NMA analyses on the "V1-TLR-2", "V1-TLR-4", "V1-TLR-5", "V2-TLR-2", "V2-TLR-4", and "V2-TLR-5" docked complexes, offering a detailed evaluation of their structural integrity and changes. The deformability graph highlights the flexible regions of the docked complexes through graphical peaks (**Figs 7A–7C and S3**). Additionally, the eigenvalue of the complexes, which indicates their structural flexibility and rigidity, showed values of 5.737061*e*–07, 5.294024*e*–07, 6.008857*e*–07, 1.209879*e*–07, 2.061794*e*–06, and 1.892946*e*–06 for the "V1-TLR-2", "V1-TLR-4", "V1-TLR-5", "V2-TLR-2", "V2-TLR-4", and "V2-TLR-5" complexes, respectively (**Figs 7D–7F and S3**). The covariance map in the NMA analysis depicts the correlated, uncorrelated, and anti-correlated movements between residues in the structures, represented by red, white, and blue colors, respectively (**Fig 7G–7I and S3**).

**Fig 8:**
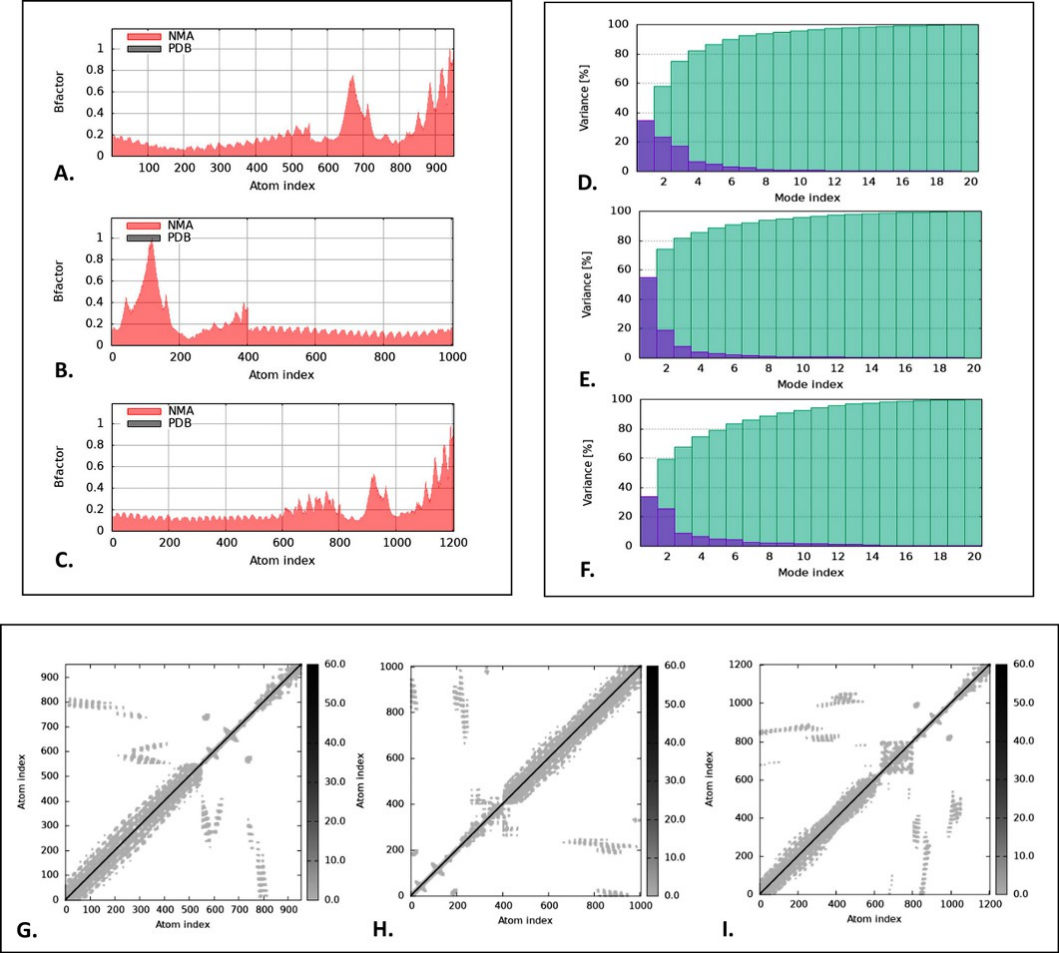
The iMODs illustration of the "V1-TLR-2", "V1-TLR-4", and "V1-TLR-5" docked complex. The illustration depicted the B-factor (**A, B, C**), variance (**D, E, F**), and elastic map (**G, H, I**).

Additionally, the B-factor graph illustrated the simulation of the docked complexes involving NMA and the PDB sector. The B-factor values represent the extent of atomic displacements, revealing higher deformability in the "V1-TLR-2", "V1-TLR-4", "V1-TLR-5", "V2-TLR-2", "V2-TLR-4", and "V2-TLR-5" docked complexes, which implies greater flexibility (**Figs 8A–8C and S4**). The variance graph displayed the cumulative variance in cyan and the individual variance in purple. In the docked complexes, the first three modes out of twenty account for 80% of the total variance (**Figs 8D–8F and S4**). The elastic map of the docked complexes, characterized by darker grey patches, indicates the interactions between atoms, suggesting the presence of more rigid regions (**Figs 8G–8I and S4**).

### 3.10 Molecular dynamic simulation

**Fig 9:**
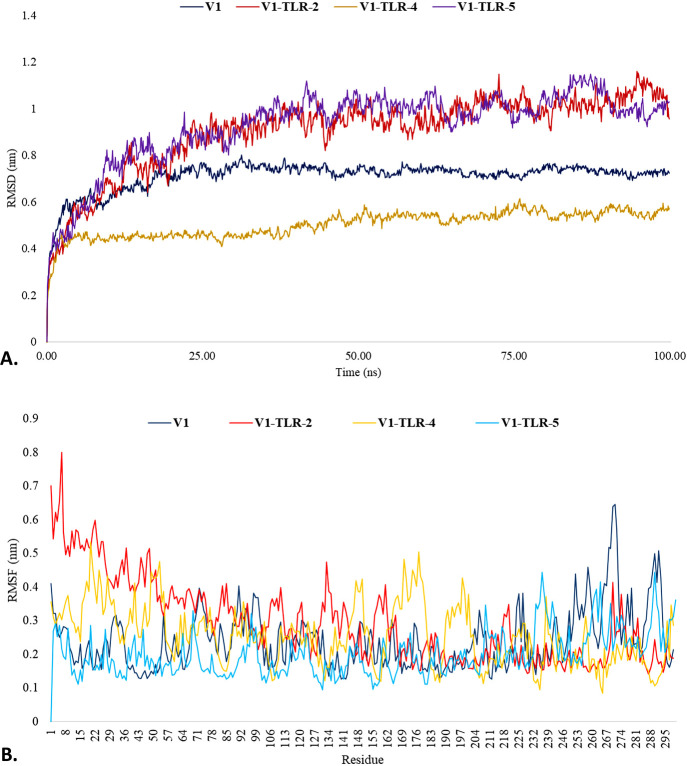
Graphical representation of the molecular dynamic simulation study. The RMSD **(A)** and RMSF **(B)** of the V1, "V1-TLR-2", "V1-TLR-4", and "V1-TLR-5" were depicted with the respective colors.

Considering the V1’s exceptional tertiary structure quality and impressive affinity for the TLRs, we decided to utilize this structure for our subsequent analysis. Therefore, The V1, "V1-TLR-2", "V1-TLR-4", and "V1-TLR-5" complexes were dynamically simulated for 100 ns to determine their stability in dynamic mode. Several assessments, including RMSD, RMSF, Rg, and SASA, were performed for MD simulation. RMSD is often employed when exploring the dynamics and structures of macromolecules. The RMSD graphic shows if the system is in equilibrium and whether the simulation length is long enough. Given the circumstances, the simulation time is sufficient for the V1, "V1-TLR-2", "V1-TLR-4", and "V1-TLR-5" complexes. This is supported by the RMSD diagram of the simulated complexes, which reached a plateau, indicating that the system has reached equilibrium. Throughout the simulation regime, the RMSD of the V1 and "V1-TLR-4" seemed to fluctuate less and remained under 1 nm, suggesting the structural stability and flexibility of the complexes. Compared to these complexes, the average RMSD of the "V1-TLR-2" and "V1-TLR-5" were more fluctuated and exceeded the acceptable value (>1 nm), suggesting the structural rigidity of the complexes. Therefore, at the end of 100ns simulation time, "V1-TLR-2" and "V1-TLR-5" showed relatively higher RMSD values than the V1 and "V1-TLR-4" complexes (**Fig 9A**). By using RMSF, the variation of the V1, "V1-TLR-2", "V1-TLR-4", and "V1-TLR-5" complexes were examined. After 100 ns simulation times, the RMSF values of these complexes were calculated to remain below 1 nm. However, the RMSF of the "V1-TLR-2" and "V1-TLR-4" fluctuated more than that of the V1 and "V1-TLR-5" throughout the simulation run (**Fig 9B**).

The Rg is a metric used to assess the density of a protein. A consistent Rg number indicates a protein’s stable folding, whereas a greater Rg profile signifies less stiffness in the biological system. The Rg values of the "V1-TLR-2" and "V1-TLR-4" complexes fluctuated steadily over simulation time. While the Rg values of the V1 and"V1-TLR-5" fluctuated abruptly, as seen in, but the values declined after 100ns simulation time (**S5A Fig**). SASA is employed in molecular dynamic simulations to forecast how much a protein’s hydrophobic core is exposed to solvents. Higher SASA values indicate that a large portion of the protein is in contact with water. In contrast, lower values suggest that most protein is inside the hydrophobic core. After 100ns, the SASA values of the V1, "V1-TLR-2", "V1-TLR-4", and "V1-TLR-5" complexes were consistent and did not exhibit any significant variations throughout the simulation, which led to a further decrease in interest (**S5B Fig**).

### 3.11 MMGBSA

For the MMGBSA analysis, we used the HawkDock server. The "V1-TLR-2" complex had scores of 590.43 kcal/mol for ΔVDWAALS, -895.88 kcal/mol for ΔEEL, 993.75 kcal/mol for ΔEGB, and -30.94 kcal/mol for ΔESURF, as revealed by the server. These interactions resulted in a total binding (ΔTOTAL) free energy of 657.36 kcal/mol (**Table 5**). The server also reported that the "V1-TLR-4" complex exhibited the following scores: -187.96 kcal/mol for ΔVDWAALS, -2397.86 kcal/mol for ΔEEL, 2499.32 kcal/mol for ΔEGB, and -24.56 kcal/mol for ΔESURF. These values resulted in a total binding (ΔTOTAL) free energy of -111.07 kcal/mol (**Table 5**). Within the "V1-TLR-5" complex, the anticipated MM-GBSA values for ΔVDWAALS, ΔEEL, ΔEGB, and ΔESURF were -243.33 kcal/mol, -1628.04 kcal/mol, 1733.78 kcal/mol, and -35.93 kcal/mol, respectively, with a total binding free energy of -173.52 kcal/mol (**Table 5**).

**Table 5:** The MMGBSA analysis of the docked complexes.

Complex	ΔVDWAALS (kcal/mol)	ΔEEL (kcal/mol)	ΔEGB (kcal/mol)	ΔESURF (kcal/mol)	ΔTOTAL (kcal/mol)
V1-TLR-2	590.43	-895.88	993.75	-30.94	657.36
V1-TLR-4	-187.96	-2397.86	2499.32	-24.56	-111.07
V1-TLR-5	-243.33	-1628.04	1733.78	-35.93	-173.52

### 3.12 MMPBSA

During this analysis, the "V1-TLR-2" complex exhibited scores of 55.83 kcal/mol for ΔVDWAALS, -733.34 kcal/mol for ΔEEL, 889.62 kcal/mol for ΔEGB, and -21.28 kcal/mol for ΔESURF. The interactions led to a total binding free energy of 122.21 kcal/mol (**Table 6**). A further report from the server indicated that the complex referred to as "V1-TLR-4" had the following scores: -211.98 kcal/mol for ΔVDWAALS, -2159.87 kcal/mol for ΔEEL, 2277.69 kcal/mol for ΔEGB, and -38.21 kcal/mol for ΔESURF. The total binding (ΔTOTAL) free energy was calculated to be -132.37 kcal/mol as a consequence of these results (**Table 6**). In the "V1-TLR-5" complex, MMPBSA values for ΔVDWAALS, ΔEEL, ΔEGB, and ΔESURF were -118.93, -1233.56, 1453.66, and -11.79 kcal/mol, respectively, with a total binding free energy of -89.38 kcal/mol (**Table 6**).

**Table 6:** The MMPBBSA analysis of the docked complexes.

Complex	ΔVDWAALS (kcal/mol)	ΔEEL (kcal/mol)	ΔEGB (kcal/mol)	ΔESURF (kcal/mol)	ΔTOTAL (kcal/mol)
V1-TLR-2	55.38	-733.34	889.62	-21.28	122.21
V1-TLR-4	-211.98	-2159.87	2277.69	-38.21	-132.37
V1-TLR-5	-118.93	-1233.56	1453.66	-11.79	-89.38

### 3.13 Codon optimization and *in silico* cloning

**Fig 10:**
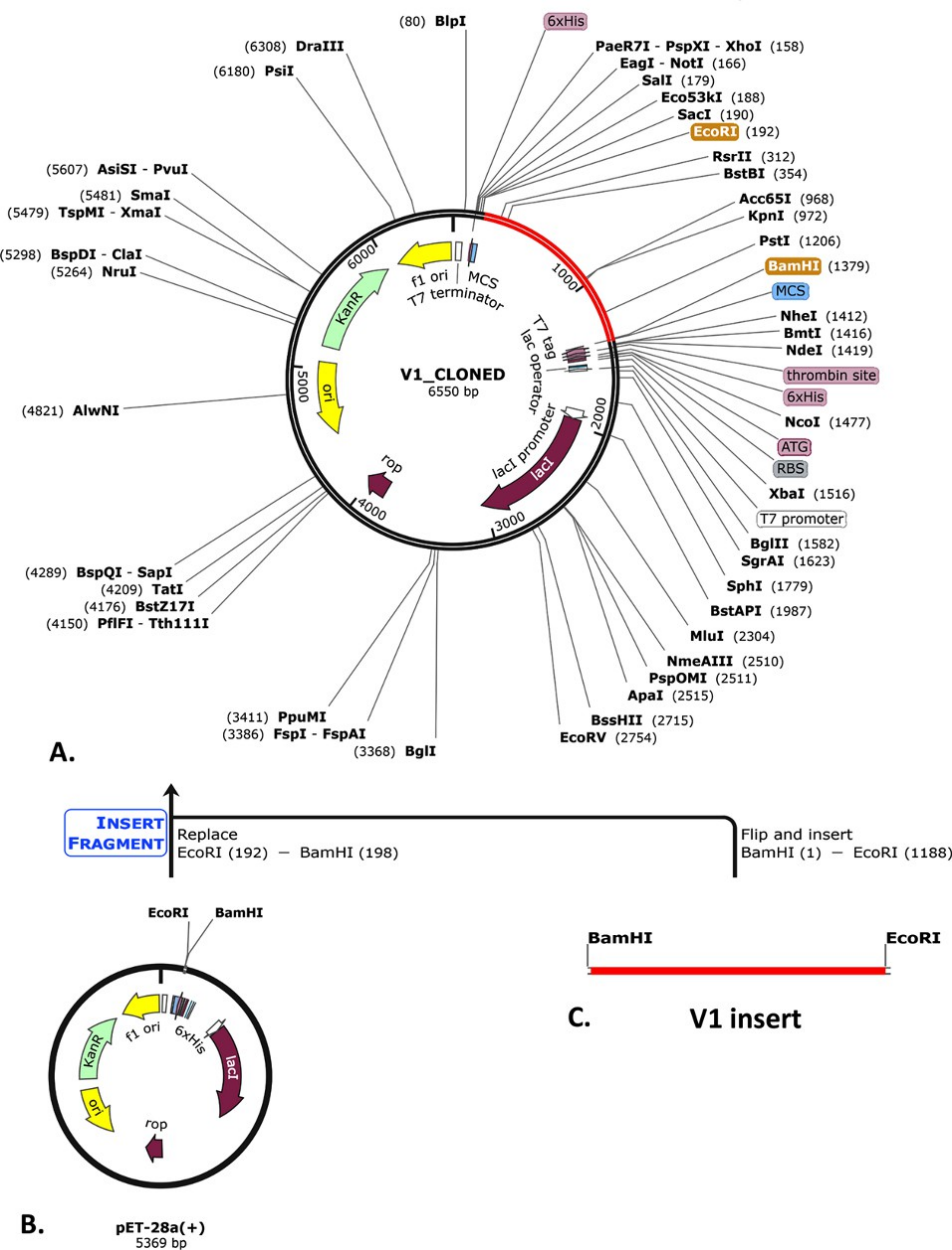
The illustration of the cloned V1 **(A)**. Codon optimization and In silico cloning of the pET-28a(+) plasmid vector **(B)** with the V1 insert **(C)**. Within the circular vector, the red part represents the V1 insert, and the black circle represents the whole vector backbone.

The codon optimization of the V1 was performed using the JCat server with *E*. *coli* strain K12. This resulted in an optimized codon sequence with 1194 nucleotide lengths (**Fig 9**). The optimized sequence’s CAI value and GC content were 0.95 and 51.59%, respectively. The optimized vaccine sequence was then inserted into the plasmid vector pET-28a(+), creating a cloned vaccine with lengths of 6550 bp (**Fig 10**).

### 3.14 Immune simulation

**Fig 11:**
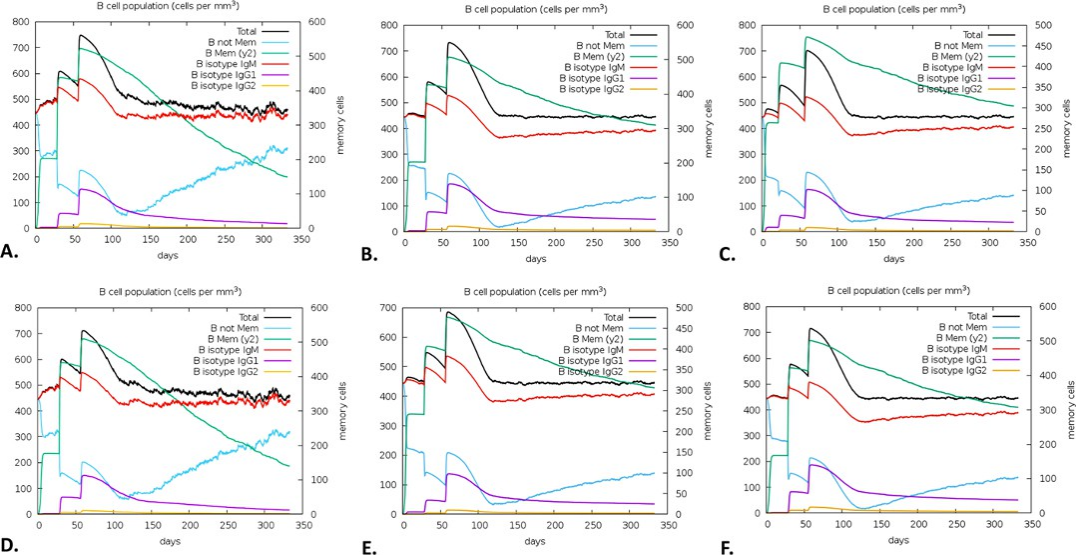
The C-ImmSim predicted the immune simulation of the V1. The evolution of B-cell among different HLA populations, including Europe **(A)**, South, West and Central Asia **(B)**, North and Central America **(C)**, Oceania **(D)**, North and East Asia **(E)**, and South America **(F).**

**Fig 12:**
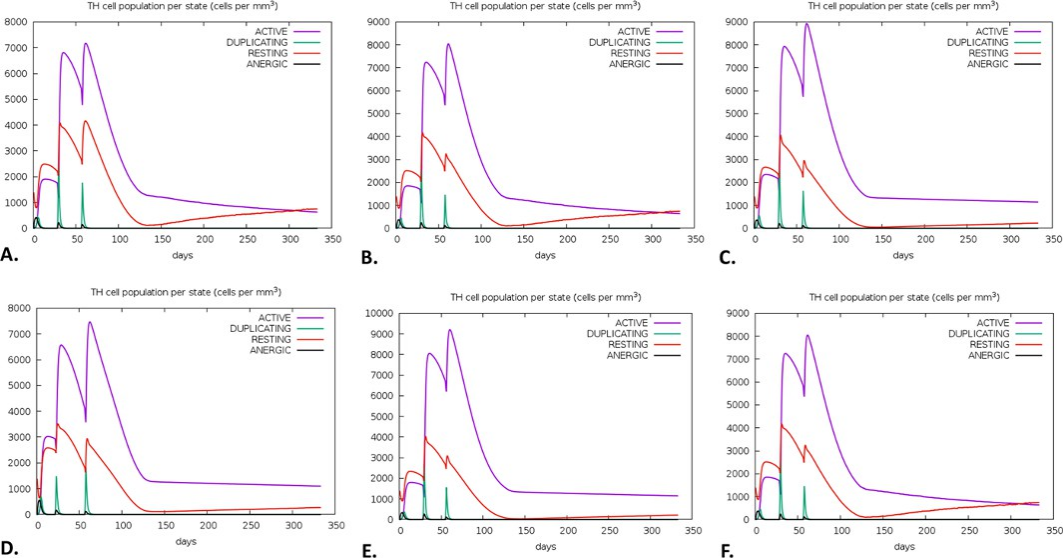
The predicted immune simulation of the V1 by the C-ImmSim server. The evolution of helper T-cell (TH) among different HLA populations, including Europe **(A)**, South, West and Central Asia **(B)**, North and Central America **(C)**, Oceania **(D)**, North and East Asia **(E)**, and South America **(F).**

**Fig 13:**
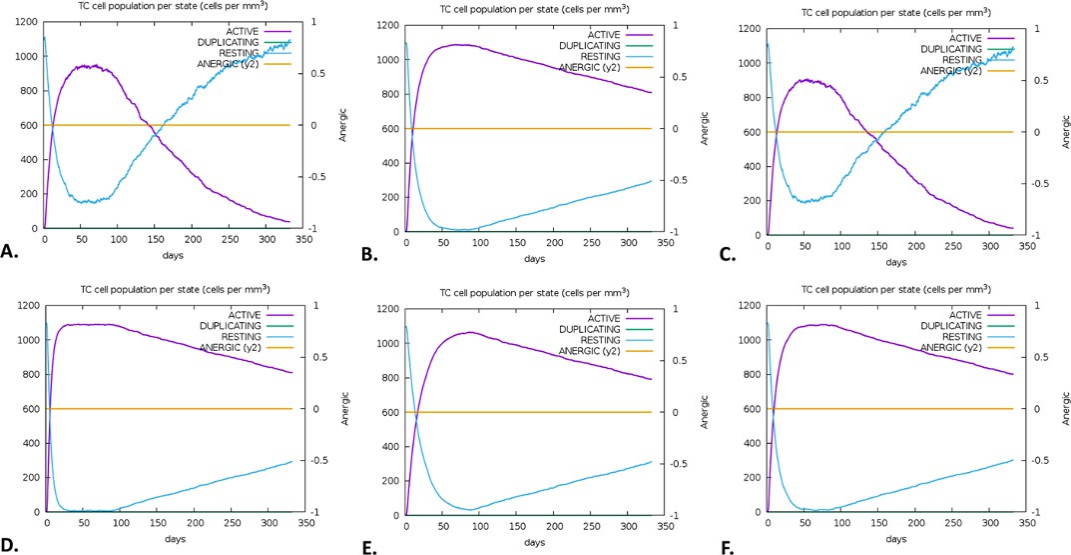
The immune simulation of the V1 by the C-ImmSim server. The evolution of cytotoxic T-cell (TC) among different HLA populations, including Europe **(A)**, South, West and Central Asia **(B)**, North and Central America **(C)**, Oceania **(D)**, North and East Asia **(E)**, and South America **(F).**

**Fig 14:**
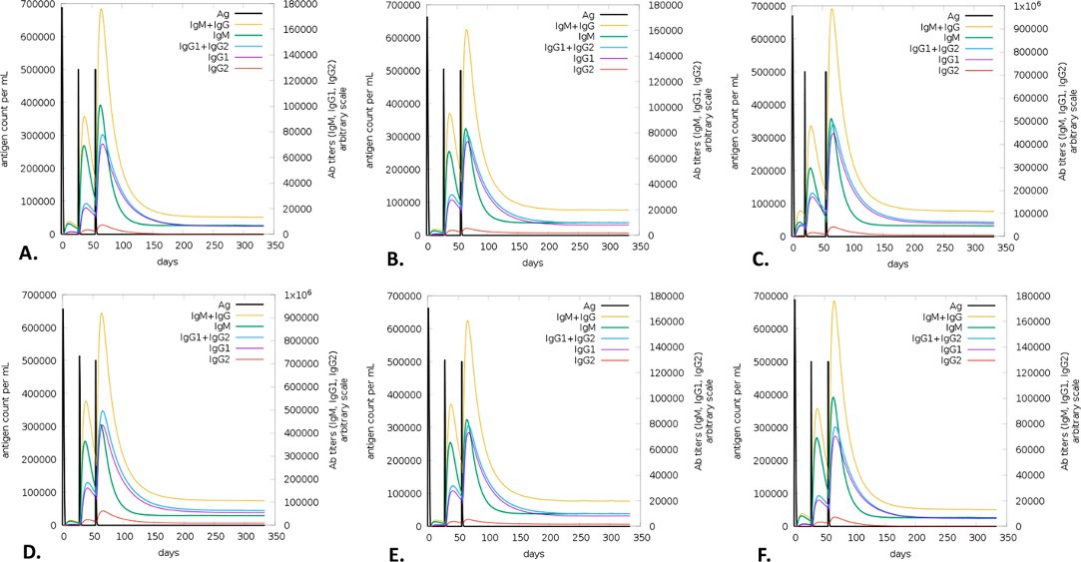
The predicted immune simulation of the V1 by the C-ImmSim server. The evolution of immunoglobulins (IgM and IgG) among different HLA populations, including Europe **(A)**, South, West and Central Asia **(B)**, North and Central America **(C)**, Oceania **(D)**, North and East Asia **(E)**, and South America **(F).**

**Fig 15:**
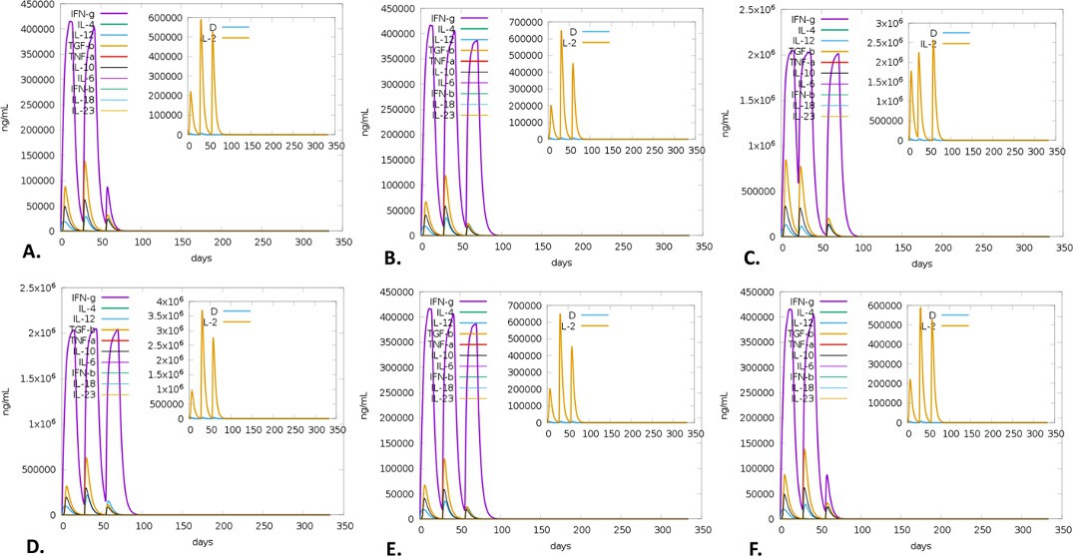
C-ImmSim server predicted immune simulation of the V1. The evolution of inflammatory cytokines among different HLA populations, including Europe **(A)**, South, West and Central Asia **(B)**, North and Central America **(C)**, Oceania **(D)**, North and East Asia **(E)**, and South America **(F).**

There was a notable increase in B-cell presence, especially memory B-cells, on the first, 28th, and 56th days following immunization. Each B-cell produced immunity that was found to be highly durable, lasting nearly a year. A high expression level indicated the early immunological responses (**Fig 11**). Furthermore, the vaccination activated T cells, specifically T helper cells (TH cells) and cytotoxic T cells (TC cells). The research also revealed that at day 60 post-vaccination, there was a notable rise in the expression of memory TH and active TH cells, which later declined over time (**Fig 12**). Furthermore, it was observed that memory cytotoxic T cell (TC) and active TC cell expression were at their peak and would persist for a very long time (**Fig 13**). Following a peak at day 60 post-vaccination, IgM+IgG expression steadily decreased, indicating that the initial immune response was solid and durable. The immune response persisted for two months after vaccination (**Fig 14**). Furthermore, the vaccination resulted in a substantial rise in the production of IFN-γ while concurrently suppressing the expression of tumor growth factor-β (TGF-β), conferring a robust immune response that persisted for two months (**Fig 15**).

## 4. Discussion

LF has been recognized as a neglected tropical disease by the WHO and the Centers for Disease Control and Prevention (CDC) [91]. Developing a more effective and durable preventive approach, such as a vaccine, is essential for controlling and stopping the spread of this parasitic infection [92, 93]. Over the past 50 years, research has been focused on developing a vaccine against LF [92, 94]. However, progress has been hindered by various factors, such as the complex life cycle of parasites, host immune responses, the lack of animal models, evidence of natural protective immunity in humans, and information on protective immune responses in humans and animals [94, 95]. Thus, this study focuses on immunoinformatics to develop a chimeric multiepitope vaccine in response to the urgent public demand for a vaccine against LF. Despite the potential, only a limited number of multiepitope vaccines against LF have been developed using this approach [24, 96–98].

Immunoinformatics is a revolutionary technology that has several advantages for developing vaccines. It relies on machine learning algorithms to rapidly sort through massive amounts of structural, genomic, and proteomic data to find potential vaccine candidates [28]. This technique enables the prediction of immune-stimulating epitopes and antigenic targets, thus enhancing the process of choosing vaccine components with improved safety and efficacy. This precision-driven approach also minimizes the need for challenging and costly clinical trials, optimizing resources and accelerating vaccine development timelines [28, 29]. Furthermore, immunoinformatics permits the development of customized vaccines that target specific pathogen strains or host populations, thereby increasing their effectiveness and adaptability in various situations. This approach promotes innovation by facilitating the investigation of novel vaccine modalities, such as nucleic acid and viral vector vaccines [18, 29]. However, several challenges must be overcome when converting immunoinformatics into vaccine manufacturing. Immunoinformatics relies on massive quantities of data, though variations in data quality and quantity may impact the accuracy of vaccine target predictions. Furthermore, predicted targets may not consistently generate the intended immune responses or may incite adverse reactions [18, 29]. The presence of a wide variety of mutations in the host population and the ongoing evolution of pathogens complicates the situation and may gradually reduce vaccine efficacy. Additional challenges include regulatory approval, production scalability, and cost-effectiveness, which require extensive resources and expertise [18, 29]. Realizing the promise of immunoinformatics in vaccine development will require interdisciplinary cooperation, technological developments, and persistent research efforts to meet these challenges. The present work used immunoinformatics approaches to identify thioredoxin and glutathione S-transferase proteins of the parasitic roundworm as prospective vaccination candidates from the pool of available vaccine antigens for *W*. *bancrofti*.

We have successfully developed two epitope-based vaccines (V1 and V2) that target the aforementioned *W*. *bancrofti* proteins (thioredoxin and glutathione S-transferase). We anticipated that the chosen MHC-1 and MHC-2 epitopes would exhibit a significant level of antigenicity without evidence of allergenicity and toxicity. Our predicted B-cell epitopes were also highly antigenic, with no evidence of allergenicity. In addition, B-cell epitopes are recognized as a crucial element in vaccine development since they significantly impact antigen-antibody interactions. We then used various linkers and adjuvants to construct in silico vaccines comprising the selected epitopes. However, the MW of the designed V1 and V2 were estimated to be 21534.46 Da and 1344.48 Da, respectively. Based on physicochemical properties, the V1 and V2 vaccines were predicted to be soluble proteins, which might prove functionally stable under body conditions [86, 99]. In this *in silico* vaccine construct, the solubility of the recombinant protein as assessed in overexpressed *E*. *coli* is essential [86]. The theoretical isoelectric point, instability index, aliphatic index, and other properties of the vaccines indicate that they are hydrophobic, consistent with reported aliphatic side chains. It is essential to comprehend how a protein folds into its secondary and tertiary structures to develop effective vaccines [100]. Subsequently, the V1 and V2 were found to have satisfactory and dependable secondary and tertiary structures. The Ramachandran plot analysis also revealed that most vaccine residues existed in the favored regions (95.6 for V1 and 96.9% for V2), indicating that the tertiary structures possessed structural integrity. The docking analyses between vaccines (V1 and V2) and human TLR-2, TLR-4, and TLR-5 using CLUSPRO 2.0 revealed the energy scores for the complexes. The lowest energy scores were observed for V1 complexes, with scores of -1248.7 (TLR-2), -1038.5 (TLR-4), and -1562.8 (TLR-5). Contrarily, V2 complexes had scores of -1004.2, -1078.5, and -1354.5, respectively. Using PyMOL and PDBsum, it was observed that V1-TLR-2 exhibited five hydrogen bonds, 19 salt bridges, and 289 non-bond interactions. Adversely, V1-TLR-4 showed four hydrogen bonds, two salt bridges, and 257 non-bond interactions. V1-TLR-5 exhibited the most significant interactions, forming many hydrogen bonds, salt bridges, and non-bond interactions. No disulfide bonds were detected in any of the complexes.

The iMODS server’s NMA of "V1-TLR" and "V2-TLR" complexes provided a complete evaluation of structural integrity and alterations. According to NMA, "V1-TLR" complexes showed more structural flexibility and rigidity than the "V2-TLR" complexes, suggested by the eigenvalues of 5.737061*e*–07 (V1-TLR-2), 5.294024*e*–07 (V1-TLR-4), 6.008857*e*–07 (V1-TLR-5), 1.209879*e*–07 (V2-TLR-2), 2.061794*e*–06 (V2-TLR-4), and 1.892946*e*–06 (V2-TLR-5). Considering its exceptional tertiary structure quality and significant binding affinity for TLRs, we used V1 for further analysis. To evaluate their stability, molecular dynamic simulations were performed on V1, V1-TLR-2, V1-TLR-4, and V1-TLR-5 complexes for 100 ns. The RMSD values suggested a state of equilibrium, with V1 and V1-TLR-4 demonstrating minimal fluctuation and high stability, measuring less than 1 nm. On the other hand, V1-TLR-2 and V1-TLR-5 exhibited higher RMSD values, suggesting a greater level of rigidity. The RMSF values stayed below 1 nm, although slightly more fluctuation was observed in V1-TLR-2 and V1-TLR-4. The Rg values fluctuated consistently for V1-TLR-2 and V1-TLR-4, while the SASA values remained constant for all complexes. Using the HawkDock server for MMGBSA analysis, total binding free energies were found to be 657.36 kcal/mol for V1-TLR-2, -111.07 kcal/mol for V1-TLR-4, and -173.52 kcal/mol for V1-TLR-5, indicating varying interaction strengths. According to MMPBSA, the "V1-TLR" complexes showed the total binding free energies were 122.21 kcal/mol for V1-TLR-2, -132.37 kcal/mol for V1-TLR-4, and -89.38 kcal/mol for V1-TLR-5. The binding affinity of V1-TLR-4 was the most significant since it exhibited the lowest free energy.

Using the JCat server for *E*. *coli* strain K12, the V1 vaccine codon was optimized to 1194 nucleotides. The optimized sequence has a CAI of 0.95 and a GC content of 51.59%, suggesting strong expression potential. Finally, a 6550-bp vaccine was cloned by inserting the optimal sequence into the plasmid vector pET-28a(+).

Additionally, we discovered a potent and long-lasting immune response following a three-dose vaccine regimen for the V1. The rise in B-cell populations and prolonged activity of both TH and TC cells suggest the possibility of long-term adaptive immunity, providing enhanced protection against *W*. *bancrofti*. Finally, this study highlights how well the vaccine against *W*. *bancrofti* elicits strong humoral and cellular immune responses. Nevertheless, this study lacks *in vitro*, *in vivo*, or clinical trials; these will be carried out in future experiments.

## 5. Conclusion

We designed two chimeric multiepitope vaccines against LF targeting the thioredoxin and glutathione S-transferase proteins of *W*. *bancrofti*. Based on physicochemical analysis reports, the designed vaccines demonstrated high antigenicity and stability with no evidence of allergenicity. Further, the vaccines showed strong binding affinities to human TLR receptors, as the docking analysis and NMA suggested. Compared to the V2, the V1 showed better efficacy and stability as a vaccine candidate. Hence, further analyses were carried out on the V1. Subsequently, the stability of V1 and its docked complexes was validated by the 100ns molecular dynamic simulation analysis. Besides, the codon optimization and in silico cloning indicated high expression levels of the V1 in *E*. *coli* plasmid vector pET-28a(+). Finally, the immune simulation revealed that a three-dose regimen of the vaccines elicited strong humoral and cellular immune responses, suggesting potential long-term protection. Nevertheless, this study has several limitations, like the absence of any *in vivo* or *in vitro* experiments with the subjects. These experiments will help to determine the vaccine’s effectiveness in preventing infection and identify potential adverse effects on human health. Therefore, future research should focus on assessing the vaccine candidate in various cell lines or animal models. We hope this study could contribute to the complete elimination of LF globally.

## Supporting information

S1 Fig Secondary structure prediction of the vaccines by GOR4 (A, B) and SOPMA (C, D) server.
(TIF)

S2 Fig The docking analysis of the "V2-TLR-2" (A), "V2-TLR-4" (B), and "V2-TLR-5" (C) complexes. The intermolecular interactions between the "V2-TLR-2" (D, G), "V2-TLR-4" (E, H), and "V2-TLR-5" (F, I) complexes are represented as different color codes. The cyan and green colors represent the TLRs and V2, respectively. The red, yellow, blue, and orange colors represent the salt bridges, disulfide, hydrogen, and non-bonded contacts, respectively.
(TIF)

S3 Fig The normal mode analysis of the "V2-TLR-2", "V2-TLR-4", and "V2-TLR-5" docked complex. The illustration depicted the deformability plots (**A, B, C**), eigenvalue (**D, E, F**), and co-variance map (**G, H, I**).
(TIF)

S4 Fig The iMODs illustration of the "V2-TLR-2", "V2-TLR-4", and "V2-TLR-5" docked complex. The illustration depicted the B-factor (**A, B, C**), variance (**D, E, F**), and elastic map (**G, H, I**).
(TIF)

S5 Fig The line plot represents the molecular dynamic simulation study. The Rg (**A**) and SASA (**B**) of the V1, "V1-TLR-2", "V1-TLR-4", and "V1-TLR-5" were depicted in different colors.
(TIF)

S1 Table Ellipro predicted the discontinuous B-cell epitopes residues of the vaccine structures. (DOCX)
