# Supplementary figures and images for: Designing of a chimeric multiepitope vaccine against bancroftian lymphatic filariasis through immunoinformatics approaches
Source: PLoS One. 2024 Sep 19;19(9):e0310398. doi: 10.1371/journal.pone.0310398 (PMC11412548; doi:10.1371/journal.pone.0310398)

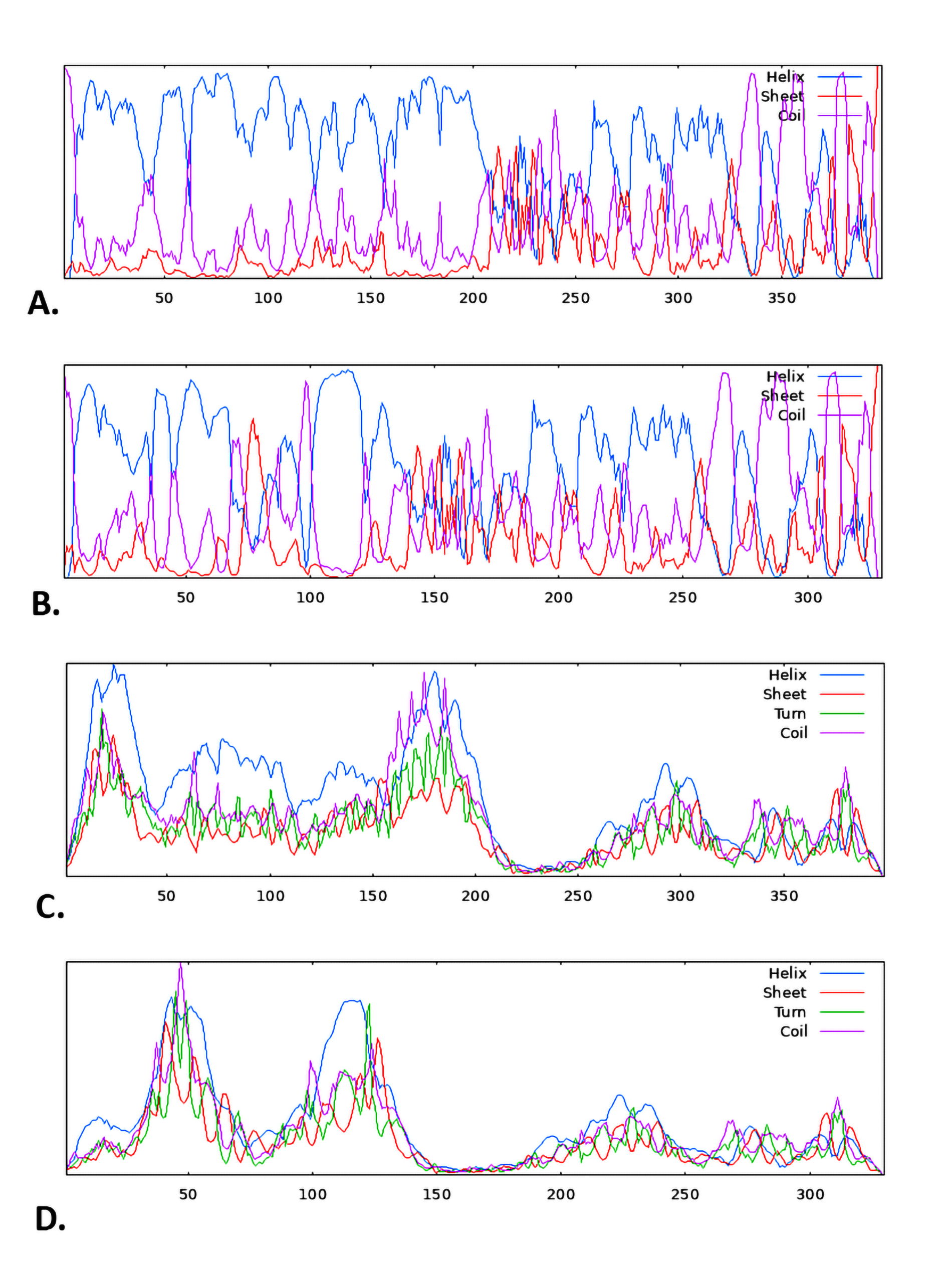

Supplement: S1 Fig — Secondary structure prediction of the vaccines by GOR4 (A, B) and SOPMA (C, D) server. (TIF) [file pone.0310398.s001.tif]

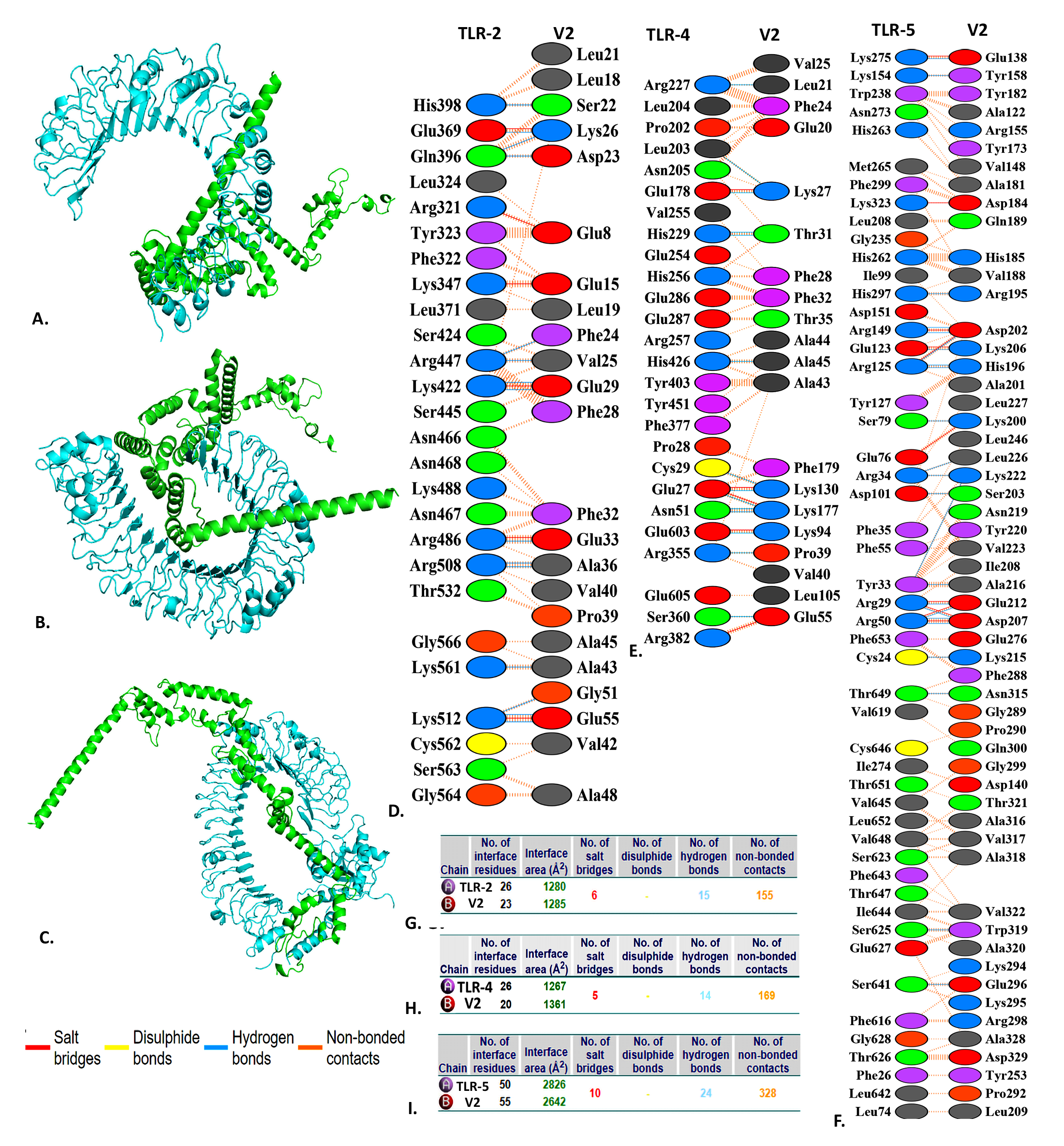

Supplement: S2 Fig — The docking analysis of the "V2-TLR-2" (A), "V2-TLR-4" (B), and "V2-TLR-5" (C) complexes. The intermolecular interactions between the "V2-TLR-2" (D, G), "V2-TLR-4" (E, H), and "V2-TLR-5" (F, I) complexes are represented as different color codes. The cyan and green colors represent the TLRs and V2, respectively. The red, yellow, blue, and orange colors represent the salt bridges, disulfide, hydrogen, and non-bonded contacts, respectively. (TIF) [file pone.0310398.s002.tif]

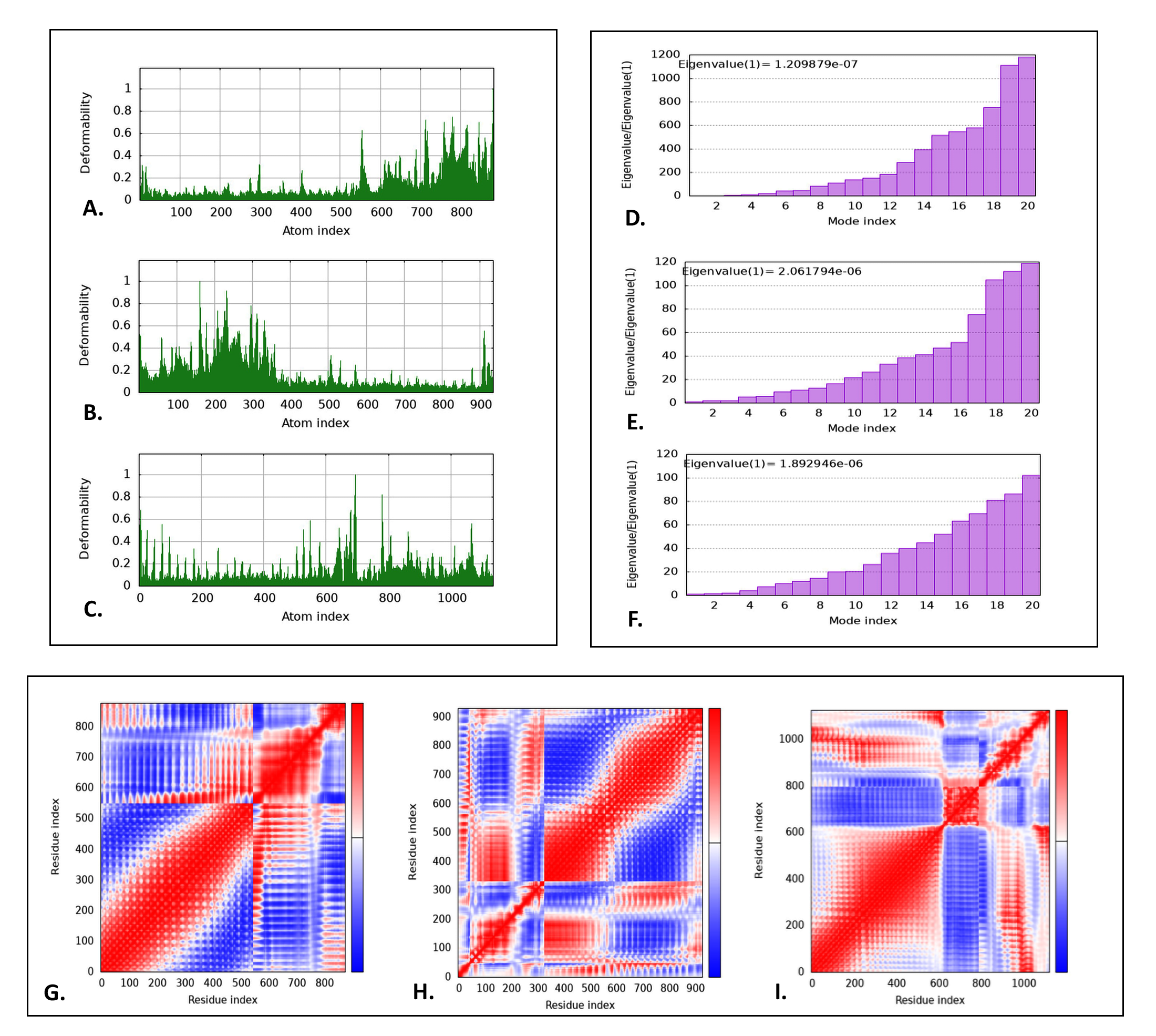

Supplement: S3 Fig — The illustration depicted the deformability plots (A, B, C), eigenvalue (D, E, F), and co-variance map (G, H, I). (TIF) [file pone.0310398.s003.tif]

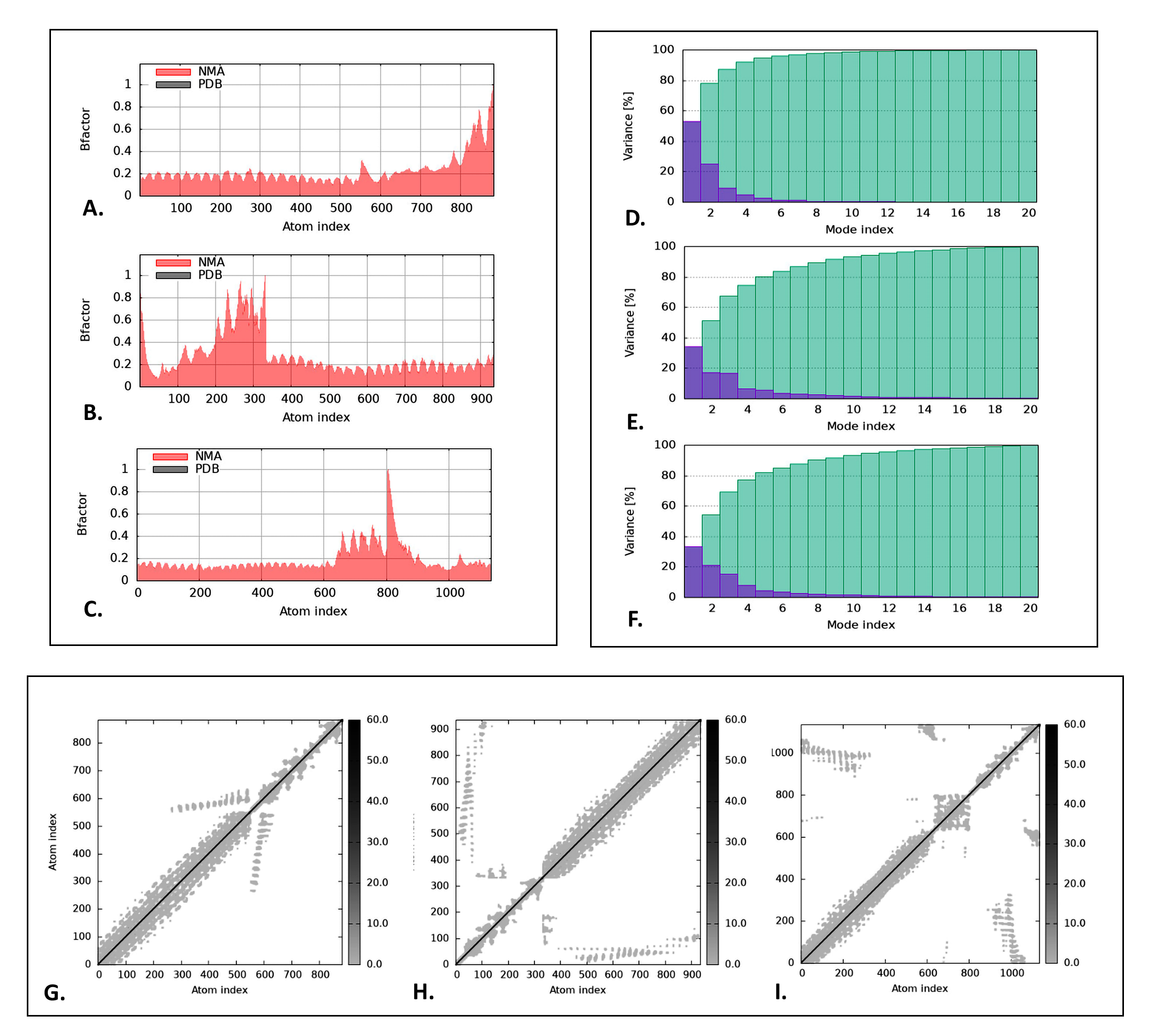

Supplement: S4 Fig — The illustration depicted the B-factor (A, B, C), variance (D, E, F), and elastic map (G, H, I). (TIF) [file pone.0310398.s004.tif]

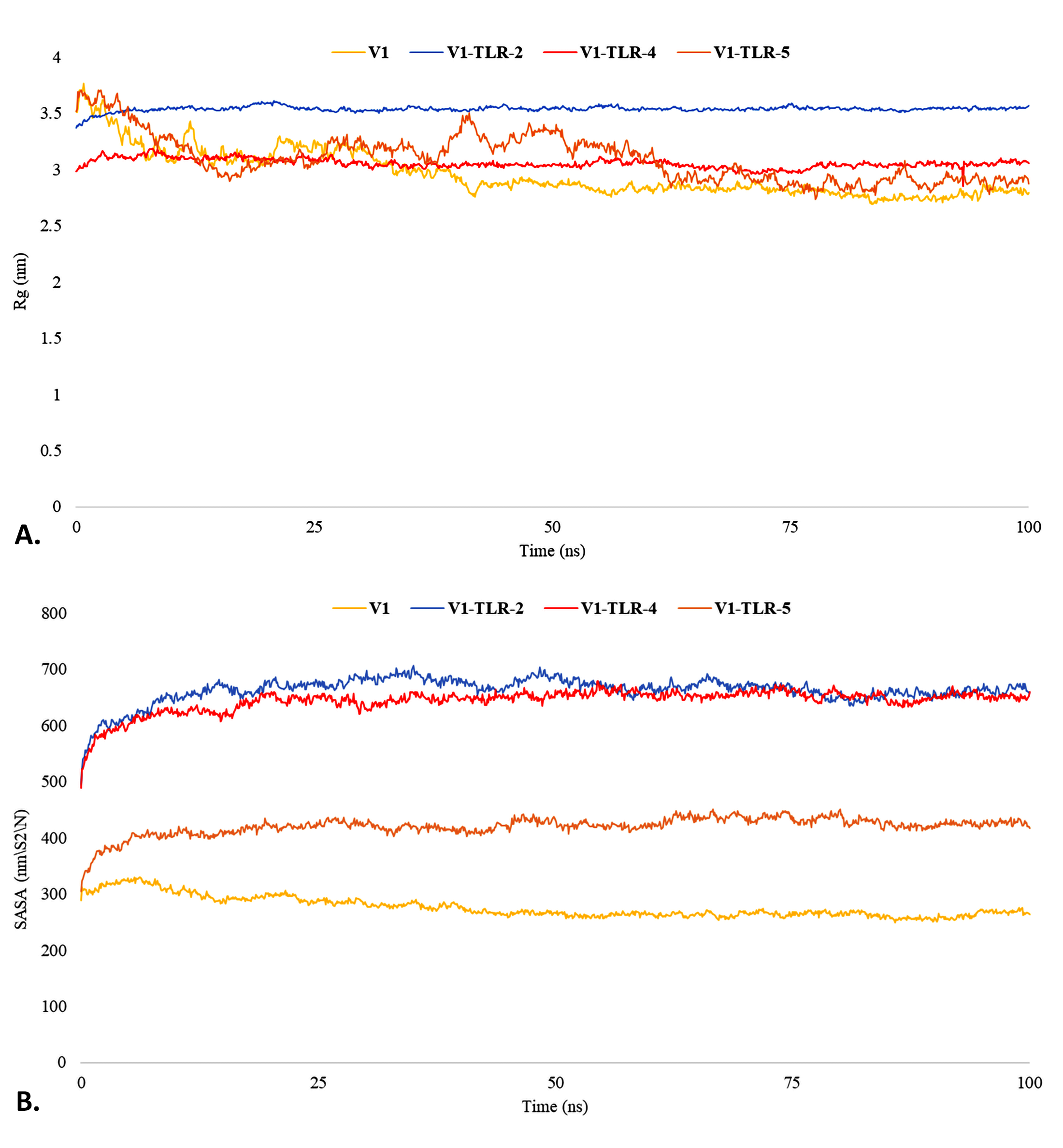

Supplement: S5 Fig — The Rg (A) and SASA (B) of the V1, "V1-TLR-2", "V1-TLR-4", and "V1-TLR-5" were depicted in different colors. (TIF) [file pone.0310398.s005.tif]
