# Supplementary material for: Designing of a chimeric multiepitope vaccine against bancroftian lymphatic filariasis through immunoinformatics approaches
Source: PLoS One. 2024 Sep 19;19(9):e0310398. doi: 10.1371/journal.pone.0310398 (PMC11412548; doi:10.1371/journal.pone.0310398)
Supplement: S1 Table — (DOCX) [file pone.0310398.s006.docx]

**S1 Table.** Ellipro predicted the discontinuous B-cell epitopes residues of the vaccine structures.

| **Vaccine** | **No** | **Residues** | **Number of residues** | **Scores** |
| --- | --- | --- | --- | --- |
| V1 | 1 | :E85, _:G86, _:L88, _:E89, _:A90, _:A91, _:T92, _:S93, _:R94, _:Y95, _:N96, _:E97, _:L98, _:V99, _:E100, _:R101, _:G102, _:E103, _:A104, _:A105, _:L106, _:E107, _:R108, _:L109, _:R110, _:S111, _:Q112, _:Q113, _:S114, _:F115, _:E116, _:E117, _:V118, _:S119, _:A120, _:R121, _:A122, _:E123, _:G124, _:Y125, _:V126, _:D127, _:Q128, _:A129, _:V130, _:E131, _:L132, _:T133, _:Q134, _:E135, _:A136, _:L137, _:G138, _:T139, _:V140, _:A141, _:S142, _:Q143, _:R145 | 59 | 0.825 |
|  | 2 | _:V28, _:N29, _:E30, _:L31, _:I32, _:T33, _:N34, _:L35, _:R36, _:E37, _:R38, _:A39, _:E40, _:E41, _:T42, _:R43, _:T44, _:D45, _:T46, _:R47, _:S48, _:R49, _:V50, _:E51, _:E52, _:S53, _:R54, _:A55, _:R56, _:L57, _:T58, _:K59, _:L60, _:Q61 | 34 | 0.798 |
|  | 3 | _:T350, _:R351, _:D352, _:D353, _:G354, _:K355, _:N356, _:F357, _:G358, _:P359, _:G360, _:P361, _:G362, _:K363, _:K364, _:E365, _:Y366, _:R367, _:G368, _:Q369, _:R370, _:S371, _:A372, _:D373, _:A374, _:L375, _:A376, _:V377, _:F378, _:G379, _:P380, _:G381, _:P382, _:G383, _:N384, _:A385, _:V386, _:A387, _:W388, _:A389, _:T390, _:V391, _:D392, _:C393, _:D394, _:R395, _:E396, _:A397, _:D398, _:I399 | 50 | 0.731 |
|  | 4 | _:A152, _:K153, _:L154, _:V155, _:G156, _:I157, _:E158, _:L159, _:P160, _:K161, _:K162, _:A163, _:A164, _:P165, _:A166, _:K167, _:K168 | 17 | 0.673 |
|  | 5 | _:K291, _:V292, _:A293, _:S294, _:L295, _:L296, _:R297, _:D298, _:D299, _:C300, _:V301, _:A302, _:K303, _:A304, _:D305, _:A306, _:L307, _:A308, _:V309 | 19 | 0.665 |
|  | 6 | _:A202, _:A203, _:D205, _:T206, _:E207 | 5 | 0.635 |
|  | 7 | _:K208, _:D209, _:S210 | 3 | 0.57 |
|  | 8 | _:A146, _:E149, _:R150 | 3 | 0.561 |
|  | 9 | _:A268, _:K269, _:A270, _:D271, _:S272, _:Y273 | 6 | 0.509 |
| V2 | 1 | _:M1, _:A2, _:K3, _:L4, _:S5, _:T6, _:D7, _:E8, _:L9, _:L10, _:D11, _:A12, _:F13, _:K14, _:E15 | 15 | 0.975 |
|  | 2 | _:M16, _:T17, _:L18, _:L19, _:E20, _:L21, _:S22, _:D23, _:F24, _:V25, _:K26, _:K27, _:F28 | 13 | 0.9 |
|  | 3 | _:M256, _:I257, _:Y258, _:Q259, _:A260, _:Y261, _:D262, _:T263, _:E264, _:K265, _:D266, _:S267, _:G268, _:P269, _:G270, _:P271, _:G272, _:A273, _:K274, _:F275, _:E276, _:K277, _:L278, _:L279, _:A280, _:T281, _:R282, _:D283, _:D284, _:G285, _:K286, _:N287, _:F288, _:G289, _:P290, _:G291, _:P292, _:G293, _:K294, _:Y297, _:R298, _:G299, _:Q300, _:R301, _:S302, _:A303, _:D304, _:A305, _:L306, _:A307, _:V308, _:F309, _:G310, _:P311, _:G312, _:P313, _:G314, _:N315, _:A316, _:V317, _:A318, _:W319, _:A320, _:T321, _:V322, _:D323, _:C324, _:D325, _:R326, _:E327, _:A328, _:D329, _:I330 | 73 | 0.757 |
|  | 4 | _:E30, _:T31, _:F32, _:E33, _:V34, _:T35, _:A36, _:A37, _:A38, _:P39, _:V40, _:A41, _:V42, _:A43, _:A44, _:A45, _:G46, _:A47, _:A48, _:P49, _:A50, _:G51, _:A52, _:A53, _:V54, _:E55, _:A56, _:A57, _:E58, _:E59, _:S61, _:F63, _:E83 | 33 | 0.623 |
|  | 5 | :L105, _:E106, _:V108, _:A109, _:E111, _:A112, _:A113, _:D114, _:E115, _:A116, _:K117, _:A118, _:K119, _:L120, _:E121, _:A122, _:A123, _:I143, _:K144, _:A145, _:Q149 | 21 | 0.618 |
|  | 6 | :K94, _:D95, _:V97, _:D98, _:G99, _:P101, _:K102 | 7 | 0.552 |
|  | 7 | :V65, _:I66, _:L67, _:E68, _:A69, _:A70 | 6 | 0.513 |
